# CK1δ/ε-mediated TDP-43 phosphorylation contributes to early motor neuron disease toxicity in amyotrophic lateral sclerosis

**DOI:** 10.1186/s40478-024-01902-z

**Published:** 2024-12-04

**Authors:** Vivian I. Ko, Kailee Ong, Deborah Y. Kwon, Xueying Li, Alicia Pietrasiewicz, James S. Harvey, Mukesh Lulla, Guruharsha Bhat, Don W. Cleveland, John M. Ravits

**Affiliations:** 1 Neuroscience Graduate Program, University of California, 9500 Gilman Drive, San Diego, La Jolla, CA 92093-0624 USA; 2 Department of Neurosciences, University of California, 9500 Gilman Drive, San Diego, La Jolla, CA 92093-0624 USA; 3 Department of Cellular and Molecular Medicine, University of California, San Diego, 9500 Gilman Drive, La Jolla, CA 92093-0624 USA; 4 Neuromuscular & Muscle Disorders, Biogen Inc., 250 Binney Street, Cambridge, MA 02142 USA; 5 Drug Metabolism and Pharmacokinetics, Biogen Inc., 250 Binney Street, Cambridge, MA 02142 USA; 6 Biotherapeutics and Medicinal Sciences, Biogen Inc., 250 Binney Street, Cambridge, MA 02142 USA

**Keywords:** Amyotrophic lateral sclerosis, Phosphorylation, TAR DNA-binding protein (TDP-43), Casein kinase 1 delta, Casein kinase 1 epsilon, Kinase inhibitors

## Abstract

Hyperphosphorylated TDP-43 aggregates in the cytoplasm of motor neurons is a neuropathological signature of amyotrophic lateral sclerosis (ALS). These aggregates have been proposed to possess a toxic disease driving role in ALS pathogenesis and progression, however, the contribution of phosphorylation to TDP-43 aggregation and ALS disease mechanisms remains poorly understood. We’ve previously shown that CK1δ and CK1ε phosphorylate TDP-43 at disease relevant sites, and that genetic reduction and chemical inhibition could reduce phosphorylated TDP-43 (pTDP-43) levels in cellular models. In this study, we advanced our findings into the hTDP-43-ΔNLS in vivo mouse model of ALS and TDP-43 proteinopathy. This mouse model possesses robust disease-relevant features of ALS, including TDP-43 nuclear depletion, cytoplasmic pTDP-43 accumulation, motor behavior deficits, and shortened survival. We tested the effect of homozygous genetic deletion of *Csnk1e* in the hTDP-43-ΔNLS mouse model and observed a delay in the formation of pTDP-43 without significant ultimate rescue of TDP-43 proteinopathy or disease progression. Homozygous genetic deletion of *Csnk1d* is lethal in mice, and we were unable to test the role of CK1δ alone. We then targeted both CK1δ and CK1ε kinases by way of CK1δ/ε-selective PF-05236216 inhibitor in the hTDP-43-ΔNLS mouse model, reasoning that inhibiting CK1ε alone would be insufficient as shown by our *Csnk1e* knockout mouse model study. Treated mice demonstrated reduced TDP-43 phosphorylation, lowered Nf-L levels, and improved survival in the intermediate stages. The soluble TDP-43 may have been more amenable to the inhibitor treatment than insoluble TDP-43. However, the treatments did not result in improved functional measurements or in overall survival. Our results demonstrate that phosphorylation contributes to neuronal toxicity and suggest CK1δ/ε inhibition in combination with other therapies targeting TDP-43 pathology could potentially provide therapeutic benefit in ALS.

**Supplementary Information:**

The online version contains supplementary material available at 10.1186/s40478-024-01902-z.

## Introduction

Amyotrophic lateral sclerosis (ALS) is a devastating and rapid fatal adult-onset neurodegenerative disease with a defining neuropathological feature of altered expression and function of transactive response DNA-binding protein 43 (TDP-43) in motor neurons of the brain and spinal cord [7, 31, 43, 48]. Specifically, TDP-43 is mislocalized from the nucleus to the cytoplasm, where TDP-43 accumulates into aggregates containing various aberrant post-translational modifications, like phosphorylation, ubiquitination, and acetylation, to name a few [8, 44, 47]. The C-terminal end of TDP-43 contains a low-complexity glycine-rich region where multiple serine residues, most notably S369, S379, S403/404, and S409/410, have been observed to be abnormally phosphorylated in ALS cases [17, 18, 22, 23]. Generally, the mechanism responsible for TDP-43 phosphorylation and its influence on the formation of cytoplasmic, aggregated, and pathological TDP-43 remains understudied, with some studies suggesting phosphorylation as a toxic mechanism [10, 19, 24, 30, 41, 54] and others supporting a protective role [6, 16, 28]. Nevertheless, phosphorylation appears to have some regulatory role for TDP-43 solubility, phase separation, and polymerization and affected TDP-43’s susceptibility for protein degradation pathways in disease states [15, 39, 40, 52, 53].

We previously identified *CSNK1E* (gene encoding CK1ε protein) as being strongly correlated with pTDP-43 pathology in ALS patient samples [27] and showed in vitro and with cellular models that CK1ε and its closely related family member CK1δ were principal kinases that regulated TDP-43 phosphorylation [26]. We demonstrated using two strategies, that genetic siRNA interference reduction and selective small molecule chemical inhibition targeting CK1δ and CK1ε reduced pTDP-43 levels effectively in both soluble and insoluble protein fractions [26]. However, cellular models remain limited and failed to capture progressive cell death over time or the complexities of mammalian biology that is observed in human patients with motor neuron degeneration as a result of ALS.

Emerging in vivo studies have investigated treatment strategies using either small molecule inhibitors or antibodies targeting S409/410 phosphorylation sites within the C-terminus of TDP-43 in various ALS-related mouse models. One group showed that treatment with an inhibitor targeting CK1δ in a TDP-43 mutation (A315T) transgenic mouse model was able to preserve motor neurons in the spinal cord and reduce glial immunoreactivity [34]. Studies which used monoclonal antibodies that targeted the C-terminal glycine-rich domain on TDP-43 in hTDP-43-ΔNLS mice showed that the treatment was able to decrease pTDP-43 and lower neurofilament light chain levels (Nf-L), a biomarker of neurodegeneration [1, 45]. However, a limitation of these studies is the lack of functional motor assays which is critical to evaluating whether these disease-modifying strategies may be suitable for advancement in preclinical therapeutic development.

In this present study, we have pursued and advanced our findings in an in vivo mammalian mouse model of TDP-43 proteinopathy (hTDP-43-ΔNLS) where mice generated cytoplasmic phosphorylated TDP-43 aggregates in neurons and exhibited significant motor behavior deficits along with shortened survival [2, 20, 50]. We tested the effects of genetic deletion of *Csnk1e* and also studied the preclinical efficacy of targeting CK1δ and CK1ε kinases by way of small molecule chemical inhibitors on TDP-43 proteinopathy biochemical and clinical behavior phenotypes in hTDP-43-ΔNLS mice. We showed that we were able to reduce TDP-43 phosphorylation and significantly lower Nf-L levels in early stages of the disease. Although this targeting was not sufficient or sustained to fully eliminate toxicity or ameliorate disease phenotypes long-term for functional benefit in this aggressive mouse model, manipulation of TDP-43 phosphorylation by selective CK1δ/ε inhibition may be a viable candidate target as a part of combinational drug therapy targeting TDP-43-pathology.

## Materials and methods

### Mouse breeding and maintenance of hTDP-43-ΔNLS mice

Bigenic transgenic mice for the hTDP-43-ΔNLS model (JAX Stock No. 028412) were generated by crossing monogenic NEFH-tTA mice (JAX Stock No. 025397) with monogenic tetO-hTDP-43-ΔNLS mice (JAX Stock No. 014650) obtained from the Jackson Laboratory (Bar Harbour, Maine, USA). Parent lines were maintained on a B6C3F1/J (JAX Stock No. 100010) background. Mice were fed with 200 mg/kg doxycycline (dox) supplemented chow (Envigo TD.08541) throughout breeding and weaning to suppress the hTDP-43-ΔNLS transgene. To induce hTDP-43-ΔNLS transgene expression in adulthood, 10-week-old mice were switched to and fed standard chow without dox (PicoLab Rodent Diet 20 #5053). Genotyping was performed using tail DNA and sent to the commercial vendor Transnetyx for automated test results. All procedures were formed in accordance with NIH Guide for the Care and Use of Experimental Animals and Studies were approved by the Institutional Animal Care and Use Committee (IACUC) of the University of California, San Diego. Littermates were randomly grouped into designated treatment cohorts in a sex-balanced way. Experimenters were blinded to the treatment cohort assignment throughout the study.

### Generation of *Csnk1e* knockout mouse line breeding with NEFH-hTDP-43ΔNLS mice

Transgenic heterozygote *Csnk1e* mutant mice were donated by Dr. David Weaver of University of Massachusetts Medical School. These mice were maintained on a C57BL/6J (JAX Stock No. 000664) background. For *Csnk1e* knockout mouse studies, *Csnk1e* mutant mice were bred with each parent line of the hTDP-43-ΔNLS mouse model, and then were subsequently crossed to create homozygous *Csnk1e*-KO x hTDP-43-ΔNLS mice. Double knockout of *Csnk1e* on both alleles was accomplished using the Cre-*loxP* system. *LoxP* sites were inserted into introns 1 and 4, where Cre-mediated excision of exons 2–3 of *Csnk1e* resulted in removal of a portion of the ATP binding domain essential for CK1ε kinase activity [12]. In the final crossing, mice were fed with 200 mg/kg doxycycline (dox) supplemented chow (Envigo TD.08541) throughout breeding and weaning to suppress the hTDP-43-ΔNLS transgene. To induce hTDP-43-ΔNLS transgene expression in adulthood, 10-week-old mice were switched to and fed standard chow without dox (PicoLab Rodent Diet 20 #5053). Genotyping was performed using tail DNA and sent to the commercial vendor Transnetyx for automated test results. All procedures were formed in accordance with NIH Guide for the Care and Use of Experimental Animals and Studies were approved by the Institutional Animal Care and Use Committee (IACUC) of the University of California, San Diego. Littermates were grouped into indicated cohorts based upon genotype in a sex-balanced way. Experimenters were blinded to genotype assignment throughout the study.

### Rotarod behavior

To assay motor coordination and balance, the rotarod (Ugo Basile) rotated at a speed of 4 rpm and accelerated to 40 rpm over 300 s. The time to fall was recorded with a maximum score of 300 s if the mouse was still running on the rod at the end of the session. There was one training session and three test sessions for each mouse with at least 30 min of rest in between sessions. The final score for each mouse was the average time of the three test sessions.

### Grip test behavior

Mice were lowered onto a triangular bar of the grip meter (Columbus Instruments) until they gripped the bar with their hindlimbs. Then, mice were pulled backwards by their tail until they released their grip and the maximum force in kilograms was recorded. One training session and five testing sessions were performed, with at least 5 min of rest in between sessions. The final score for each mouse was an average of the five test sessions.

### Compound muscle action potential (CMAP) recordings

Mice were anesthetized by inhalation of 2.5% isoflurane. Once completely anesthetized (checked by pinching toes for reflexes), mice were immobilized on a heated operating table. Animals were placed prone on the table, and the backs and legs were shaved with professional hair clippers and cleaned with ethanol and Betadine. Each leg was positioned at a 45-degree angle from the body and the feet were taped down using surgical tape to prevent any excess motion that could have affected the recordings. Disposable monopolar needle electrodes (28-gauge TECA model 902-DMF25-TP) were used for stimulation and recording. All five electrodes used were connected to the appropriate port of the Natus XLTEK XCalibur LT machine. The sciatic nerve was stimulated using an electrode pair and current was delivered using a stimulating cathode placed subdermally at the sciatic notch, roughly 8 mm lateral from the midline and 2 cm anterior to the base of the tail. The anode was placed subdermally roughly 4 mm lateral from the midline. The reference electrode was placed 3–4 mm subdermally above the ankle near the tendons. The recording electrode was subdermally placed on the surface of the middle of the tibialis anterior (TA) muscle of either the right or left leg (both legs were recorded for each subject). The ground electrode was placed near the midline subdermally. Each animal was stimulated with incremental pulse intensity for 0.1 ms beginning at 1.0 mA and increasing in 0.5 mA steps until the CMAP amplitude stopped increasing (maximum current 5 mA). Once the maximum amplitude was identified, 5 recordings were taken at this supra-maximal stimulus intensity. Responses were recorded separately for both the left and right TA muscles of each mouse with the left hind limb recorded before the right hind limb and analysis for each mouse was an average of the right and left hind limb measurements.

### Neurofilament-light (Nf-L) measures

Mice were anesthetized by isoflurane vapors in a glass chamber box and toe pinch was used to test depth of anesthesia. Once anesthetized, scissors were used to decapitate the mouse, and the trunk blood was poured into K2 EDTA tubes (BD #365974). To separate plasma from blood, samples were spun at 11,000 rpm for 5 min at 4 °C and the supernatant was saved in a fresh Eppendorf tube and stored at -80 °C until use. Plasma samples were sent to collaborators at Biogen Inc. for Nf-L measurements. Nf-L was measured in plasma samples using the ProteinSimple Nf-L cartridge V5 kit according to the manufacturer’s guidelines. In brief, plasma samples were brought to room temperature and diluted 1:6 in Sample Diluent buffer. Samples were mixed and 50 µL volumes were transferred to wells within the Ella cartridge. One milliliter of Wash Buffer A was added to each designated well of the Ella cartridge and the cartridge was run on the Ella machine using the Simple Plex Runner software.

### Pharmacokinetic characterization

The concentration-time profile of CK1δ/ε-specific PF-05236216 inhibitor and CK1ε-specific PF-4,800,567 inhibitor in whole brain and plasma were analyzed in B6C3F1/J (JAX Stock No. 100010) mice after a single intraperitoneal (IP) injection. The compound was injected at either a dose of 40 mg/kg (for CK1δ/ε-specific PF-05236216 inhibitor) or 100 mg/kg (for CK1ε-specific PF-4800567 inhibitor) in vehicle solution of 10% DMSO with 90% of 20% hydroxypropyl β-cyclodextrin in 0.9% saline. The dosing volume for the compound was 2 mg/mL. Mice were weighed prior to injection to ensure appropriate volume was administered. Samples were collected at 1, 2, 4, 8, and 24 h after dosing (*n* = 6, with 3 females and 3 males for each time point). Mice were anesthetized by isoflurane vapors in a glass chamber box and toe pinch was used to test depth of anesthesia. Once anesthetized, scissors were used to decapitate the mouse, and the trunk blood was poured into K2 EDTA tubes (BD #365974) and brain was quickly extracted into 4.5 mL TallPrep Lysing matrix tubes (MP Biomedical #116973100). Whole brain tissues were snap frozen and stored at -80 °C until use. To separate plasma from blood, samples were spun at 11,000 rpm for 5 min at 4 °C and the supernatant was saved in a 96-well plate and stored at -80 °C until use.

### LC/MS for compound detection

Whole brain tissue and plasma samples were sent to collaborators at Biogen Inc. for pharmacokinetic compound exposure measurements. Generally, for protein extraction via precipitation method, a 1 µg/mL (1000 ng/mL) stock solution was first prepared in DMSO for each compound, either CK1δ/ε-specific PF-05236216 inhibitor or CK1ε-specific PF-4,800,567 inhibitor. A calibration curve was constructed using a 12-point serial dilution scheme with final concentrations 1000 ng/mL down to 0.100 ng/mL. Brains were homogenized at 5×, with 1:4 of tissue:1× PBS (w/v). Aliquots of 10 µL of each sample, standard, and matrix blanks were added to designated wells in a 96-well extraction plate. Matrix matching was done to normalize all samples 1:1, plasma: brain homogenate, with a total extraction volume of 20 µL. Double blanks were precipitated with 6× volume of acetonitrile. All other wells were precipitated with 6x volume of internal standard (25 ng/mL glyburide, 50 ng/mL chrysin and carbutamide in acetonitrile). Samples were vortexed for 30 s then centrifuged at 3500 rpm for 5 min. Next, 50 µL of supernatant was transferred to an empty 96-well injection plate and diluted with 50 µL of water. Samples were vortexed gently, then plate was capped and placed into the autosampler for injection into the mass spectrometer (Triple Quad 5500, AB Sciex Instruments). The LC-MS/MS analytical conditions used were Ace 3 C18-PFP (2.1 × 50 mm) for the column, 0.1% formic acid in water for mobile phase A, 0.1% formic acid in acetonitrile for mobile phase B, 0.1% formic acid in (80:10:10 H2O: ACN: MeOH) for needle rinse 1 port, 0.1% formic acid in (50:50 ACN: MeOH) for needle rinse 2 pump, and 50 µL loop volume.

### Tissue collection for biochemistry assays

Mice were anesthetized by isoflurane vapors in a glass chamber box. Toe pinch was used to test depth of anesthesia. Once anesthetized, scissors were used to decapitate the mouse, and then the brain and spinal cord tissues were extracted and partitioned into 1.5 mL Eppendorf tubes. Once tissues were collected, tubes were flash frozen onto dry ice and stored in -80 °C until use.

### Tissue collection for histology assays

Mice were injected via intraperitoneal (IP) with avertin, and toe pinch was used to test depth of anesthesia. Once anesthetized, mice were perfused with freshly prepared ice-cold PBS and 4% paraformaldehyde (PFA) using a peristaltic pump. When sufficiently fixed, whole brain and spinal cord tissues were extracted and put into conical tubes containing 4% PFA for overnight fixation at 4 °C. The following day, the solution was changed to 30% sucrose. Once the tissue was saturated with sucrose, it was embedded into OCT blocks for cryosectioning at the desired thickness (35 μm) and stored in cryoprotectant solution until use for immunofluorescent staining.

### Real-time PCR (qRT-PCR)

RNA was extracted using the Direct-zol RNA miniprep kit (Bio-Rad) according to the manufacturer’s protocol and samples were measured for RNA quantity (OD260/280) and purity (OD230/260) using a NanoDrop spectrophotometer (Thermo Fisher). qRT-PCR was performed with iTaq universal SYBR Green one-step kit (Bio-Rad) and CFX384 (Bio-Rad) as described in the manufacturer’s protocol. Each 10 µL reaction contained iTaq universal SYBR Green reaction mix (2×), iScript reverse transcriptase, forward and reverse primers (300 nM), RNA (10 ng/µL), and nuclease-free H_2_O. The PCR reactions were carried out as follows: 10 minutes at 50°C for the reverse transcription reaction, 1 minute at 95°C for the polymerase activation and initial DNA denaturation followed by 40 cycles of amplification at 95°C for 10 seconds and 60°C for 30 seconds. The primer sequences for genes detected were *Mus musculus* casein kinase 1 delta (*Csnk1d*, NM_027874, forward 5’- CTGAGGGTCGGGAACAGGTA − 3’, reverse 5’- TGAGGATGTTTGGTTTTGACACA − 3’), *Mus musculus* casein kinase 1 epsilon (*Csnk1e*, NM_013767, primer set 1 forward 5’- AAGCTCGAATGTGTGAAGACG − 3’, primer set 1 reverse 5’- TGACCATCACGTTATAGTCTCCC − 3’, primer set 2 forward 5’- AGGTTGAAGCATGGAGTTGC − 3’, primer set 2 reverse 5’- TGGGGATGTTTCGTCTTCAC − 3’), *Mus musculus* TAR DNA binding protein (*Tardbp*, NM_145556, forward 5’- ATGAGAACGATGAACCCATTGAA − 3’, reverse 5’- TGAGACACGGGATTCCGGTAG − 3’), *Homo sapiens* TAR DNA binding protein (*TARDBP*, NM_007375, forward 5’- CCGAACAGGACCTGAAAGAG − 3’, reverse 5’- CAGTCACACCATCGTCCATC − 3’), and *Mus musculus* glyceraldehyde-3-phosphate dehydrogenase (*Gapdh*, internal standard, NM_008084, forward 5’- AGGTCGGTGTGAACGGATTTG − 3’, reverse 5’- TGTAGACCATGTAGTTGAGGTCA − 3’).

### RNA-sequencing and gene expression analysis

RNA was extracted using the Direct-zol RNA miniprep kit (Bio-Rad) according to the manufacturer’s protocol and samples were measured for RNA quantity (OD260/280) and purity (OD230/260) using a NanoDrop spectrophotometer (Thermo Fisher). The RNA was sent to UCSD Institute for Genomic Medicine (IGM) Genomics Center for sample QC, library prep, and sequencing. RNA-seq library was constructed using the Illumina Stranded Total RNA Prep with Ribo-Zero Plus kit (Illumina) according to manufacturer’s protocol. The sequencing was completed using the Illumina NovaSeq X Plus system. For analysis of the transcriptomic data, reads were aligned to UCSC genome browser *mus musculus* (mouse) genome mm39 using STAR alignment tool with standard parameters. The aligned reads were quantified to the ensemble transcripts release 110 annotation model using standard parameters. Genes with more than 20 counts were filtered for and then normalized. DESeq2 was used to analyze for differentially expressed genes and filtered for a fold change (FC) <-2 or > 2 and a false discovery rate (FDR) < 0.05. Gene set enrichment analysis (GSEA) was performed according to standard protocols.

### Protein extraction

To characterize pTDP-43 expression in samples, frontotemporal cortex brain tissue was homogenized in ice-cold N-PER buffer (Thermo Fisher) with protease and phosphatase inhibitors (Thermo Fisher) and then incubated on ice for 10 min. Samples were then centrifuged at 13,500 rpm for 20 min at 4 °C and the supernatant was saved in a new tube as the soluble fraction. The pellet was washed twice with N-PER buffer to remove residual soluble protein. The pellet was resuspended in lysis buffer + 8 M urea + 3% SDS and then sonicated, followed by incubation on ice for 15 min, then centrifuged at 14,000 rpm for 10 min at 10 °C. The supernatant was then saved as the insoluble protein fraction.

### Immunoprecipitation (IP)

SureBeads Magnetic Beads (BioRad) were vortexed in their solution to resuspend, and 100 uL was transferred into a tube. Beads were magnetized and the supernatant was discarded. Beads were then washed 3 times with 1 mL PBST (PBS + 0.1% Tween-20) by vortexing and magnetizing to discard the supernatant. The IP antibody was then diluted in PBST and added to the beads for resuspension. Antibodies used were CK1δ (Thermo Fisher #MA5-17243) and CK1ε (Thermo Fisher #MA5-517231). The mixture was rotated at room temperature for 10 min, then vortexed, magnetized, and the supernatant was discarded. The beads (with antibody bound to them at this stage) were washed 3 times with 1 mL PBST and then protein lysate (with protease and phosphatase inhibitors, Thermo Fisher) was added to incubate with beads and antibody for rotation at room temperature for 1 h. After the incubation, the beads were washed 3 times with PBST by vortexing, magnetizing, and discarding the supernatant. The protein was eluted into 1× orange loading buffer by incubation at 70 °C for 10 min. Beads were then magnetized, and the eluent was moved to a new vial for immunoblotting.

### Immunoblotting

To determine protein sample concentrations, Pierce BCA Protein Assay (Thermo Fisher) was used. Samples were loaded in equal volumes and normalized to 10 µg with PBS and then boiled with 4× Protein Loading Buffer (LI-COR) + β-mercaptoethanol for 5 min at 90 °C. Samples were then loaded onto a 4–15% SDS‒PAGE gel to run (80 V for 10 min, then 100 V for 50 min), then transferred to PVDF membranes (85 V for 60 min on ice) using a Bio-Rad Mini-Protean Tetra Cell Electrophoresis System. Membranes were blocked using TBS Intercept blocking buffer (LI-COR) and then incubated with primary antibodies diluted in Intercept T20 Antibody Diluent (LI-COR) overnight at 4 °C. The primary antibodies used were pTDP-43 (S409/410) (1:1000, Proteintech #22309-1-AP), TDP-43 (1:500, Proteintech #12892-1-AP), CK1δ (1:1000, Thermo Fisher #MA5-17243), CK1ε (1:1000, Thermo Fisher #MA5-517231), β-actin (1:20,000, Proteintech #66009-1-Ig), and GAPDH (1:20,000, Proteintech #60004-1-Ig). The following day, the membranes were washed with TBST (0.1% Tween-20 in TBS) three times for 10 min each at room temperature and then incubated with secondary antibodies (1:20,000 IRDye 800CW and 1:20,000 IRDye 680RD, LI-COR) diluted in Intercept T20 Antibody Diluent (LI-COR) for 1 h at room temperature. Membranes were then washed 3 times with TBST for 10 min each and then 2 times with TBS for 10 min each before imaging on the Odyssey^®^ Fc Imaging System (LI-COR). ImageStudioLite was used to analyze signal intensity. Quantification of band intensity was based upon immunoblot densitometry values. The pTDP-43 band signal was first normalized to β-Actin, as was the total TDP-43 band signal. These two normalized values were then computed as a ratio with pTDP-43 relative to total TDP-43. This analysis incorporated normalization not only to an internal protein control within each sample using the housekeeping gene β-Actin, but also considered the expression of the total TDP-43 (hTDP-43-ΔNLS transgene) since there may have been inherent genetic regulatory variability between samples. Raw western blot images are included in Supplementary file 2.

### Immunofluorescence (IF)

Tissue sections were removed from cryoprotectant solution and washed in PBS 3 times for 10 min each. Sections were then blocked in 5% horse serum with 0.2% TritonX in PBS for 1 h at room temperature. Tissue sections were then incubated overnight at 4 °C with the primary antibodies diluted in blocking buffer. The primary antibodies used for staining were TDP-43 (1:500, BioLegend #808301) and pTDP-43 (S409/410) (1:500, Proteintech #22309-1-AP). The following day, tissues were washed three times with PBS for 10 min each and then incubated with fluorescently labeled secondary antibodies (Alexa Fluor 488 and 594, Thermo Fisher) diluted at 1:500 in blocking buffer for 2 h at room temperature. Tissues were then washed twice with PBS for 10 min each and stained with DAPI at 1:1000 for 5 min at room temperature. Tissues were washed in PBS three times for 10 min each and then mounted with ProLong Gold Antifade Mountant with DAPI (Invitrogen). Slides were left at room temperature to dry completely before imaging. Imaging was performed using the UCSD Microscopy Core’s Zeiss LSM 880 Confocal with FAST Airyscan at 20x and 63x with oil.

### Statistical analyses

Data were analyzed using GraphPad Prism10 and are expressed as the mean ± standard error of the mean (SEM). Immunoblotting analysis was performed by normalizing bands of interest to the signal intensity for the internal sample control β-actin, and then a ratio for pTDP-43 (S409/410) over total TDP-43 levels was calculated and reported as a relative intensity measure. Statistics were performed with either two-way ANOVA or Mann-Whitney U test using GraphPad Prism 10 with p-values less than 0.03 considered significant.

## Results

### hTDP-43-ΔNLS mouse model of inducible TDP-43 proteinopathy has ALS disease-relevant phenotypes

**Fig. 1:**
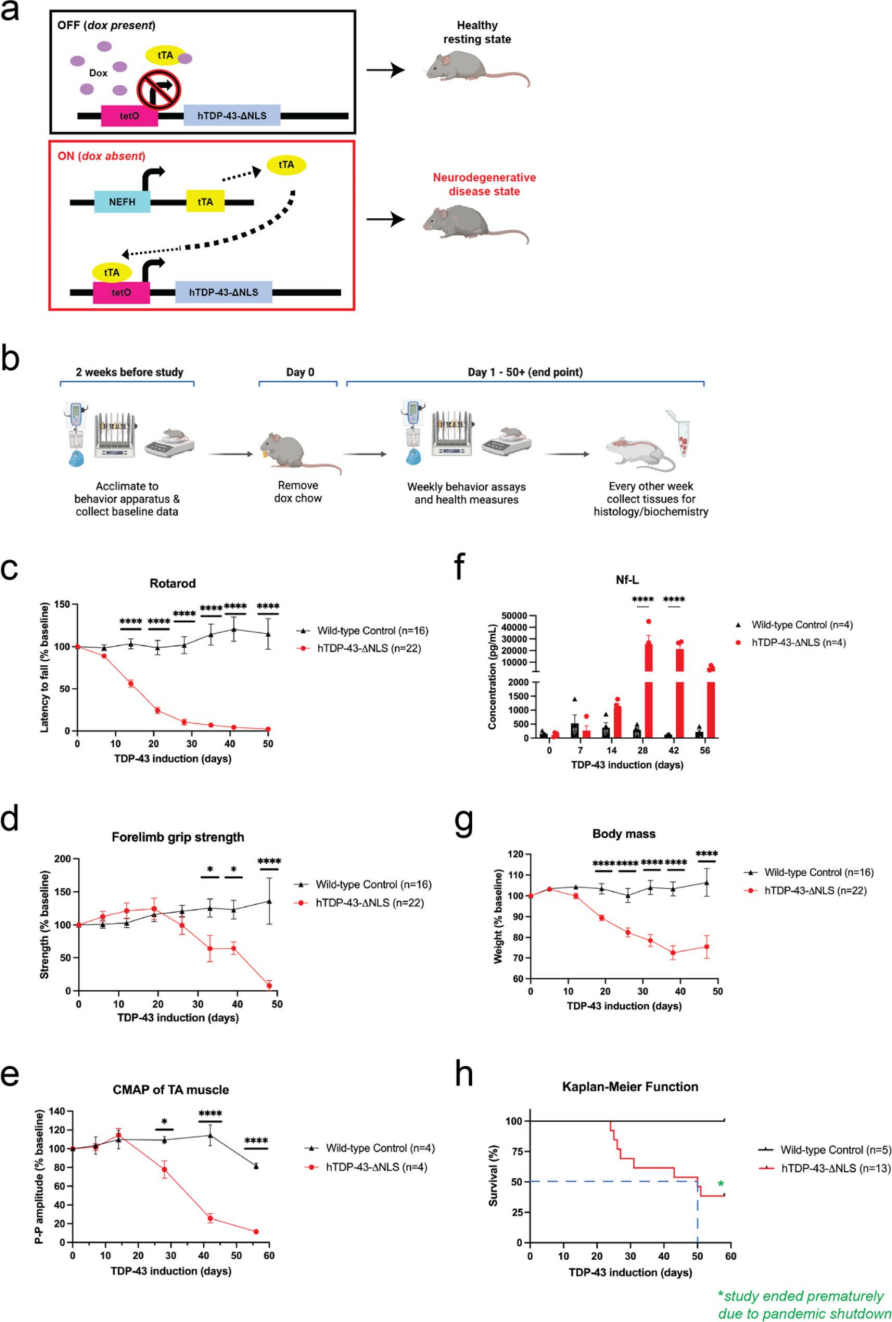
hTDP-43-ΔNLS mouse model is a tet-off dox inducible system with significant motor deficits and neurodegenerative health. (**a**) When hTDP-43-ΔNLS mice were fed dox chow, the hTDP-43-ΔNLS transgene was suppressed, and mice grew normally into adulthood. The neurofilament heavy chain (NEFH) promoter line drove cell-specific expression in neurons with large caliber axons, which were primarily motor neurons of the brain and spinal cord. When dox chow was replaced with normal chow, the tetracycline activator protein (tTA) activated the transcription of the hTDP-43-ΔNLS transgene, which encoded the human TARDBP gene that contained a defective nuclear localization signal (ΔNLS). This initiated the progressive TDP-43 proteinopathy disease phenotypes and neurodegeneration in mice. (**b**) Experimental design used to characterize and validate the hTDP-43-ΔNLS mouse model. Mice were trained on behavior tasks in the weeks prior to the study start date, and the baseline data was collected before dox chow was changed to regular chow. Data on motor behavior and health measures was collected weekly. Satellite mice were run in parallel for tissue collection to have samples that represented different stages of disease progression. (**c**) The cohort for rotarod, grip strength test, and body mass included 16 wild-type control mice (9 male, 7 female) and 22 hTDP-43-ΔNLS mice (13 male, 9 female). Rotarod performance for hTDP-43-ΔNLS mice was significantly decreased by 14 days. (**d**) Forelimb grip test strength declined significantly by 32 days. (**e**) The cohort for CMAP and Nf-L included 4 wild-type control mice (2 male, 2 female) and 4 hTDP-43-ΔNLS mice (2 male, 2 female). CMAP recordings showed significant decline in the peak-to-peak amplitude in TA muscles by day 25 of induced TDP-43 expression. (**f**) hTDP-43-ΔNLS mice displayed elevated Nf-L levels by 14 days of induced TDP-43 expression, with significant increases by day 28. (**g**) Body mass quickly declined as disease progressed in hTDP-43-ΔNLS mice. (**h**) The cohort for survival included 5 wild-type control mice (3 male, 2 female) and 13 hTDP-43-ΔNLS mice (8 male, 5 female). The median survival was reached by day 50, as indicated by the blue dashed lines. The study ended prematurely in 2020 due to the COVID pandemic shutdown. Two-way ANOVA with repeated measures and Dunnett’s multiple comparisons, *p < 0.03, **p < 0.002, ***p < 0.0002, ****p < 0.0001. Error bars represent SEM

To translate our previous studies into a relevant mammalian disease model, we used hTDP-43-ΔNLS mice, which is a tet-off doxycycline (dox) inducible system with overexpression of human TDP-43 containing a defective nuclear localization signal (NLS) (Fig. 1a). When dox is removed during adulthood at 10 weeks old, allowing transgene expression, mice develop hallmark ALS phenotypes of motor neuron degeneration in brain and spinal cord, motor behavior deficits, declining health measures, and premature death with median survival of 8–10 weeks [2, 20, 50]. In our study to comprehensively characterize and validate the disease mouse model (Fig. 1b), we found that the TDP-43 transgene expressed was over 50-fold higher than the endogenous TDP-43 (Supplementary figure S1f-g) and observable symptom onset of motor deficits for rotarod and forelimb grip strength tasks were significant by 14 days and 32 days of induced TDP-43 expression, respectively, as compared to littermate wild-type control mice (Fig. 1c-d). We also conducted compound muscle action potentials (CMAP) recordings for both right and left hindlimb tibialis anterior (TA) muscles via stimulation of the sciatic nerve as a proxy for muscle neurophysiology and motor neuron degeneration [35]. The CMAP recordings showed significant decline in peak-to-peak amplitude by day 25 of induced TDP-43 expression (Fig. 1e).

**Fig. 2:**
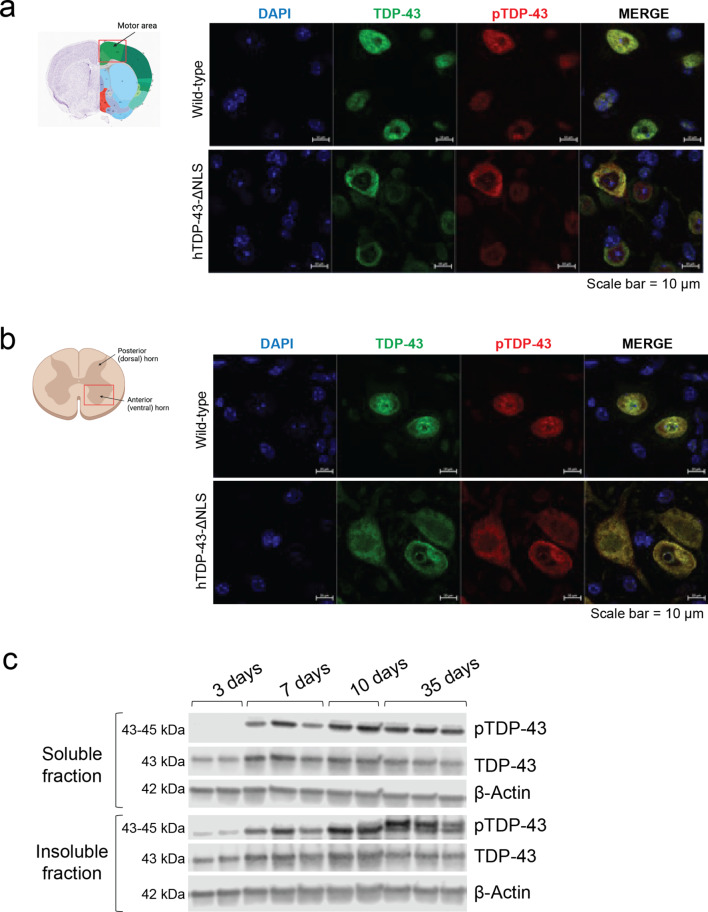
pTDP-43 accumulates in the cytoplasm of neurons and is detected before observable motor deficits in hTDP-43-ΔNLS mice. (**a**) Representative immunofluorescence images of neurons in cortex of wild-type control and hTDP-43-ΔNLS mice with staining for TDP-43 (green), pTDP-43 (red), and DAPI (blue). TDP-43 remained nuclear in wild-type cells while it became translocated and mislocalized to the cytoplasm in hTDP-43-ΔNLS samples. Much of the mislocalized TDP-43 was also found to be phosphorylated. Scale bars represent 10 μm. Overview of anatomical section was obtained from the Allen Mouse Brain Atlas, https://mouse.brain-map.org. (**b**) Similar observations of altered TDP-43 expression were also observed in lumbar spinal cord tissue sections. Scale bars represent 10 μm. (**c**) Representative immunoblots demonstrate that pTDP-43 was detected in soluble and insoluble protein fractions after 7 days of induced TDP-43 expression, with maximal signal intensity by day 10. pTDP-43 accumulated and was detected in the insoluble fraction before it could be detected in the soluble fraction. Later into the disease course, a higher molecular weight hyperphosphorylated TDP-43 species (top band) could be detected in the insoluble fraction

Neurofilament light chain (Nf-L) level is a common early-stage biomarker of neurodegeneration and neuronal damage, and is increasingly used as a biomarker of therapeutic efficacy in clinical trials, which can be measured in biofluids like CSF or blood plasma [4, 5]. Increases in Nf-L levels has been shown to be correlated with neurodegenerative disease progression. We found that the Nf-L level in this mouse model was increased by day 14 and significantly elevated by day 28 of induced TDP-43 expression (Fig. 1f). Additionally, their body mass significantly declined throughout disease progression, and the median survival was reached by day 50 (Fig. 1g-h). At the cellular level, there was distinct TDP-43 nuclear clearance and striking cytoplasmic phosphorylated TDP-43 (pTDP-43) expression, as detected with an antibody for the S409/410 phosphosites, in neurons of both brain and spinal cord (Fig. 2a-b). The pTDP-43 antibody (ProteinTech 22309-1-AP) used may not have been as specific as we would ideally want, but it provided the best signal compared to 7 other pTDP-43 antibodies tested which all failed (ProteinTech 66079-1-Ig, CosmoBio TIP-PTD-P05, CosmoBio TIP-PTD-P07, CosmoBio TDP-PTD-M01, ProteinTech 66318-1-Ig, ProteinTech 80007-1-RR, BioLegend 829901), The nuclear signal for pTDP-43 is difficult to understand but may be due to technical aspects of the antibody, acknowledging the complexity of nucleo-cytoplasmic shuttling mechanisms of TDP-43. However, the immunoblots (further explained below) provided the confidence that pTDP-43 was expressed in this mouse model ((Fig. 2c). These phenotypes of the hTDP-43-ΔNLS mouse model, which reflected the ALS disease progression in patients and human postmortem neuropathological observations, enabled us to study the effect of CK1δ/ε-mediated phosphorylation on TDP-43 proteinopathy in vivo.

### TDP-43 hyperphosphorylation and cytoplasmic accumulation precedes motor behavior deficits in hTDP-43-ΔNLS mice

Previous characterization and studies of hTDP-43-ΔNLS mice has not established the temporal dynamics of TDP-43 phosphorylation and accumulation once the transgene is activated. Thus, we characterized this in both soluble and insoluble protein fractions via detection in immunoblots. We found that pTDP-43 was detected 7 days after TDP-43 induction in both soluble and insoluble protein fractions, with maximal signal intensity by day 10 (Fig. 2c). This data indicated that TDP-43 phosphorylation preceded motor deficits, which were observable at day 14 of induced TDP-43 expression. The insoluble protein fraction contained detectable pTDP-43 before the soluble protein fraction, suggesting phosphorylation may be a modification that occurred after insoluble TDP-43 formation. Further, accumulation of a higher molecular weight (> 43 kDa) hyperphosphorylated TDP-43 species was detected exclusively in the insoluble protein fraction at later timepoints (Fig. 2c).

These biochemical observations support phosphorylation as a mechanism that occurred earlier in disease pathogenesis before behavioral symptoms could be detected, allowing us a window of approximately 10 days to examine how CK1δ- and CK1ε-mediated phosphorylation would affect pTDP-43 accumulation and protein solubility during disease progression. Additionally, this model sufficiently generated an altered higher molecular weight insoluble hyperphosphorylated TDP-43 species later in disease progression, which was reflective of the neuropathological observations of TDP-43 protein recovered from human samples [17] and allowed us to study the short- and long-term effects of CK1δ- and CK1ε-mediated phosphorylation on disease progression.

### Deletion of *Csnk1e* does not alter the regulation of *Csnk1d*

Studies of the CK1 kinase family showed that CK1δ and CK1ε shared significant homology in their kinase domains which suggested potential redundant roles, depending on the biological pathway [9, 13, 14]. Genetic deletion studies in mice revealed that CK1ε may be dispensable, but CK1δ was indispensable and a critical component for cell viability since homozygous deletion was perinatal lethal [12], so we were unable to pursue studies of *Csnk1d* via genetic knockout in our hTDP-43-ΔNLS disease mouse model.

To test whether removal of the *Csnk1e* gene would affect TDP-43 phosphorylation in vivo, we generated homozygous *Csnk1e* knockout (KO) in the hTDP-43-ΔNLS mouse model. We pursued genetic KO due to confidence in complete reduction of the gene, contrary to the incomplete and potentially insufficient knockdown with ASO or siRNA strategies that could confound our results and data interpretation. Homozygous *Csnk1e*-KO mice displayed no behavioral deficits or overt phenotypes and bred normally. To produce homozygous *Csnk1e*-KO x hTDP-43-ΔNLS mice, homozygous *Csnk1e*-KO mice were bred with each parent line of the hTDP-43-ΔNLS mouse model which were the hemizygous NEFH-tTA mice and hemizygous tetO-hTDP-43-ΔNLS mice. Offspring with the desired genotypes were then crossed to create homozygous *Csnk1e*-KO x hTDP-43-ΔNLS mice, which will be referred to as *Csnk1e*-KO mice onwards (Supplementary figure S1a). The homozygous *Csnk1e*-WT x hTDP-43-ΔNLS littermate controls will be referred to as *Csnk1e*-WT moving forward. In this final crossing, mating pairs were fed dox chow to suppress transgene expression so pups could develop properly and grow into adulthood. The homozygous knockout of *Csnk1e* on both alleles was accomplished using the Cre-*loxP* recombination system, where *LoxP* sites were inserted into introns 1 and 4 and Cre-mediated excision of exons 2–3 of *Csnk1e* resulted in deletion of a part of the ATP binding domain essential for CK1ε kinase activity [12]. Due to Mendelian genetics where only 6.25% of offspring in our breeding scheme would have the desired genotypes, over 450 mice were bred to achieve the necessary sample size for experiments.

Given that *Csnk1d* shared 85% homology with *Csnk1e* by comparing their nucleotide sequences using the Basic Local Alignment Search Tool (BLAST) on NCBI (https://blast.ncbi.nlm.nih.gov/Blast.cgi), we validated and characterized the *Csnk1e*-KO and *Csnk1e*-WT mice for genetic deletion specificity and potential compensatory gene regulation. Additionally, BLAST alignment comparisons revealed that human *CSNK1D* and *CSNK1E* shared 98% homology with respective mouse *Csnk1d* and *Csnk1e*, thus the results from our study in mice would likely be relevant and applicable to human contexts.

Homozygous deletion of *Csnk1e* was found to be gene-specific, without significant changes to *Csnk1d* RNA expression when compared to *Csnk1e*-WT littermate controls (Supplementary figure S1b-c). Furthermore, immunoblots of immunoprecipitated samples for the low expressing CK1ε and CK1δ kinases showed lack of CK1ε protein in *Csnk1e*-KO mice, while CK1δ was still detected at similar levels to controls (Supplementary figure S1d-e). The CK1 antibodies used for immunoprecipitation and immunoblotting were validated using recombinant proteins and found to be specific for solely either CK1δ or CK1ε, not both, providing confidence that there was no observable significant compensatory *Csnk1d* expression in *Csnk1e*-KO mice (data not shown). In addition, the RNA expressions of both endogenous mouse *Tardbp* gene and the overexpressed human *TARDBP* transgene in *Csnk1e*-KO mice indicated no significant change compared to *Csnk1e*-WT controls (Supplementary figure S1f-g). These characterizations and validations allowed the results of our study to be attributed to the specificity of the *Csnk1e* knockout.

### Deletion of *Csnk1e* delayed pTDP-43 formation, but was insufficient in reducing TDP-43 proteinopathy long-term

**Fig. 3:**
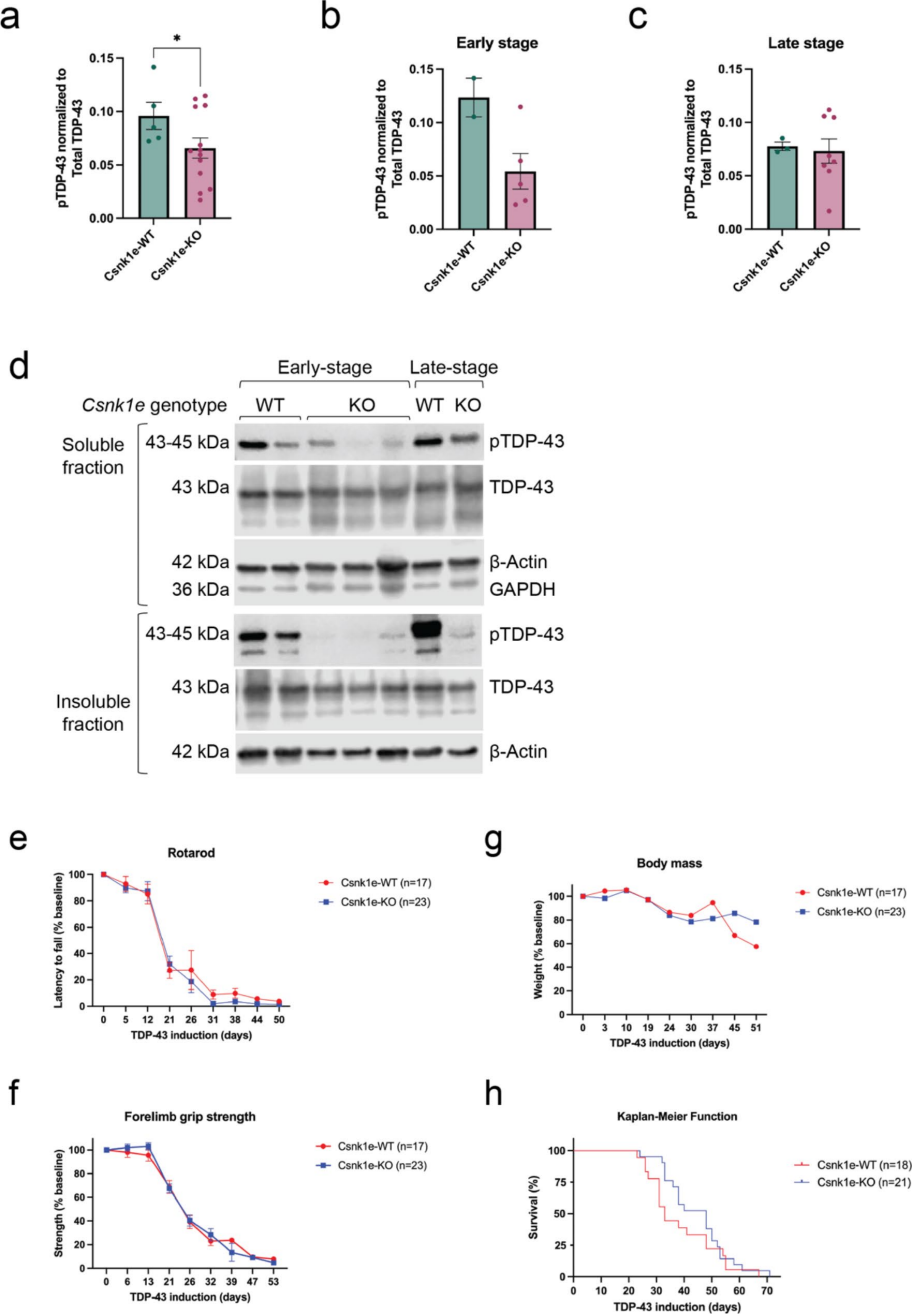
Deletion of *Csnk1e* delayed pTDP-43 formation but did not improve motor behavior on hTDP-43-ΔNLS mice. (**a**) *Csnk1e*-KO mice had generally reduced pTDP-43 levels in soluble protein fraction across all timepoints collected. (**b**-**c**) Data from panel 3a was grouped into early (less than 21 days) and late (more than 21 days) stages of disease progression. Only samples from early stage *Csnk1e*-KO mice demonstrated pTDP-43 reduction. Quantification of pTDP-43 reduction in soluble protein fraction was computed as a normalized ratio between pTDP-43 and total TDP-43 signals. The signal intensity for each pTDP-43 band was first normalized to β-Actin, then calculated as a ratio relative to total TDP-43 levels (which was also normalized to β-Actin). Mann-Whitney U test, *p < 0.03, **p < 0.002, ***p < 0.0002, ****p < 0.0001. Error bars represent SEM. (**d**) Fractionating the protein into soluble and insoluble compartments demonstrated a delay in soluble pTDP-43 formation in *Csnk1e*-KO mice from early to late stage. GAPDH signal was used to indicate the quality of fractionation between insoluble and soluble protein fractions. (**e**) The cohort for rotarod, forelimb grip strength test, and body mass included 17 Csnk1e-WT mice (5 male, 12 female) and 23 Csnk1e-KO mice (12 male, 11 female). Rotarod performance for *Csnk1e*-KO mice did not improve as compared to *Csnk1e*-WT control mice. (**f**) Forelimb grip strength test also did not display improvements. (**g**) *Csnk1e* deletion did not exacerbate the overall health of mice, and the body mass declined similarly in both groups. (**h**) The cohort for survival included 18 Csnk1e-WT mice (10 male, 8 female) and 21 Csnk1e-KO mice (10 male, 11 female). There was a modest extension of median survival temporarily for *Csnk1e*-KO mice. Two-way ANOVA with repeated measures and Dunnett’s multiple comparisons, *p < 0.03, **p < 0.002, ***p < 0.0002, ****p < 0.0001. Error bars represent SEM

Following the same experimental design as discussed above (Fig. 1b), we assayed the mice for general health measures and motor behavior tests until mice reached a natural humane endpoint, which was 71 days for the study, and then analyzed tissue samples afterwards. Biochemical analysis of soluble protein fraction showed that *Csnk1e*-KO mice had generally reduced pTDP-43 levels across all timepoints collected (Fig. 3a). We then explored possible trends by grouping into early stage (less than 21 days) and late stage (more than 21 days) of disease progression based upon the onset of motor deficits and declining function (Fig. 1c-h). Though available sample size was not sufficient for statistical testing, this analysis suggested that *Csnk1e* may be a driver of TDP-43 phosphorylation, and its absence may have decreased pTDP-43 in the early stages in this mouse model, but that this effect was not sustained in later stages where pTDP-43 levels of *Csnk1e*-KO mice were similar to that of *Csnk1e*-WT control mice (Fig. 3b-c).

Because we detected a difference in pTDP-43 levels depending on whether *Csnk1e*-KO mice were early or late stage, we pursued protein fractionation of frontotemporal cortex tissue into soluble and insoluble fractions for further analysis. The immunoblots suggested a delay in both soluble and insoluble pTDP-43 formation from early to late stage, where the late-stage mice had comparable soluble pTDP-43 levels as controls (Fig. 3d). The emergence of pTDP-43 levels while CK1ε was no longer present may be due to other kinases, like CK1δ or others, that could phosphorylate TDP-43. More interestingly, we observed that pTDP-43 was not detected in the insoluble protein fractions either in the early and late stages in the *Csnk1e*-KO mice. Due to limited sample availability, statistical analysis was not possible. However, these striking trends in our results suggest that deletion of *Csnk1e* may have delayed pTDP-43 formation. Further, they suggest that phosphorylation of TDP-43 may actually be a mechanism that promotes protein solubility and drives TDP-43 into a soluble state (Fig. 3d).

In our motor behavior studies, we did not observe an improvement on either rotarod or forelimb grip strength tests (Fig. 3e-f) and the *Csnk1e*-KO group performed similarly as the control *Csnk1e*-WT group. Body mass yielded no significant difference between groups, which also indicated that *Csnk1e* deletion was not detrimental to accelerate or worsen the overall health of mice (Fig. 3g). Analyzing the tissues collected for immunohistochemistry and fluorescent confocal imaging revealed that neurons of *Csnk1e*-KO mice still developed hallmarks of TDP-43 proteinopathy with TDP-43 nuclear clearance and cytoplasmic pTDP-43 expression in the brain and spinal cord (Supplementary figure S2a-b). However, we observed a brief period between days 25 to 45 where the Kaplan-Meier curve suggested an extension of survival (Fig. 3h), and this seems consistent with the reduced and delayed pTDP-43 formation (Fig. 3b and d). Despite this temporary extension of survival, there was no accompanied overall change in reducing or slowing disease symptoms or progression. Both *Csnk1e*-KO and *Csnk1e*-WT groups reached their end points in a similar amount of time. Thus, we concluded *Csnk1e*-KO impacted progression early in disease but was overall insufficient in reducing hallmark phenotypes of TDP-43 proteinopathy in the hTDP-43-ΔNLS mouse model in the long-term.

### Characterization of CK1 inhibitors as an in vivo tool

We previously used CK1δ/ε-specific PF-05236216 inhibitor and CK1ε-specific PF-4,800,567 inhibitor in vitro to demonstrate their efficacy in reducing pTDP-43 levels in U2OS and SH-SY5Y cell models that generated phosphorylated TDP-43 [26] (Supplementary Table S1). In order to optimize using these inhibitors for reliable in vivo experiments, we conducted early dose-finding studies to characterize the pharmacokinetic (PK) and pharmacodynamic (PD) properties for both CK1 inhibitors in mice and identify the inhibitor dose and dosing route and regimen. These studies allowed us to explore the exposure-response relationship between compound exposure concentrations in the brain and the compound’s target engagement effectiveness and toxicity. We identified the maximal tolerated dose (MTD) for CK1δ/ε-specific PF-05236216 inhibitor was 80 mg/kg from the range of doses we tested at 20, 40, 80, 120 mg/kg based upon survival outcomes post-injection. We did not encounter a MTD for CK1ε-specific PF-4,800,567 inhibitor with the range of doses we tested at 25, 50, 100, and 150 mg/kg.

**Fig. 4:**
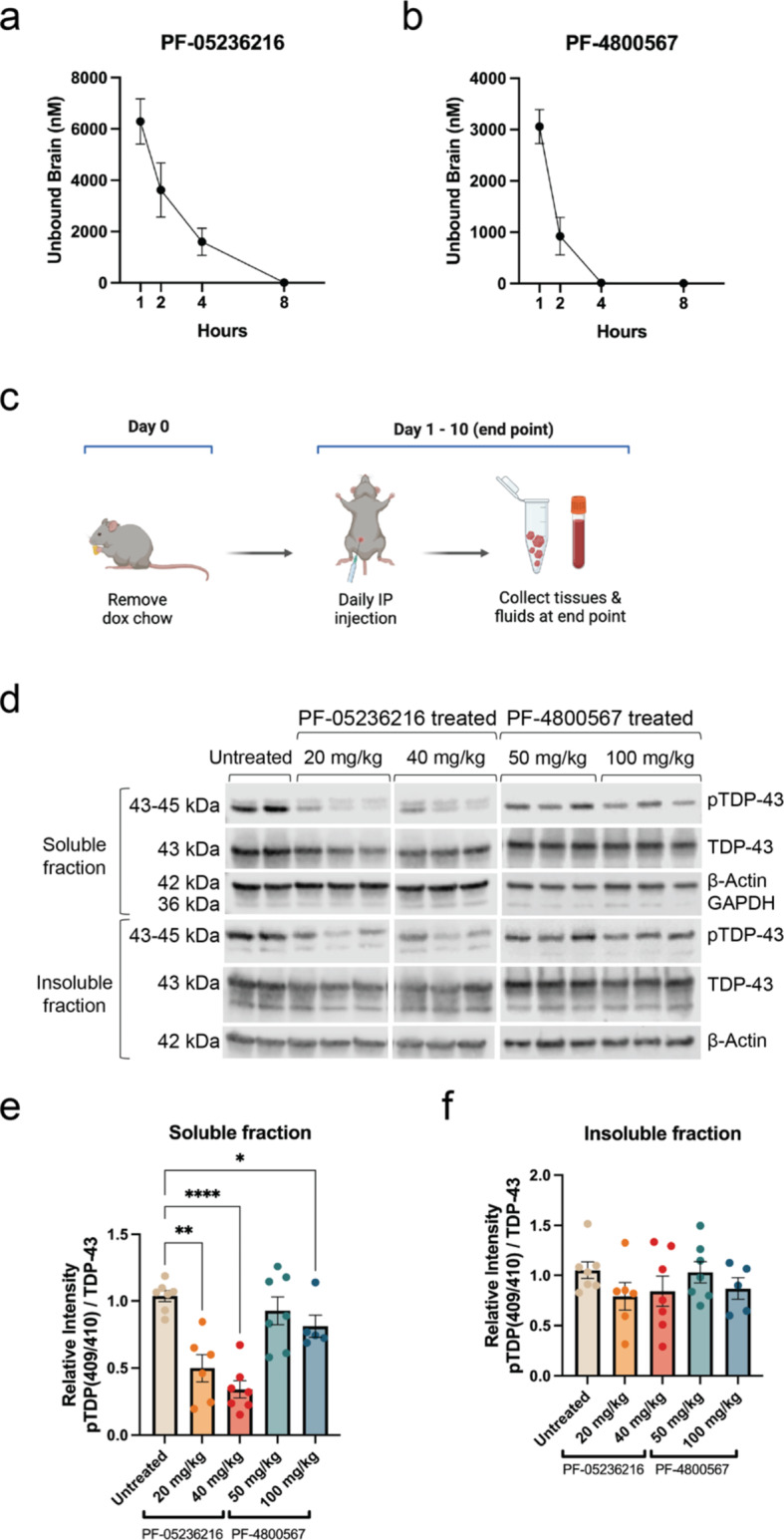
Pharmacokinetic (PK) and pharmacodynamic (PD) characterization of CK1 inhibitors in vivo. (**a**) The PK profile for 40 mg/kg dose of CK1δ/ε-specific PF-05236216 inhibitor measured as unbound compound concentrations in the brain demonstrated a half-life approximately 4–6 h long. (**b**) The PK profile for 100 mg/kg dose of CK1ε-specific PF-4,800,567 inhibitor measured as unbound compound concentrations in the brain demonstrated a half-life of approximately 1–2 h long. Samples were collected 1-, 2-, 4-, 8-, and 24-hours after a single IP injection. Group sizes were *n* = 6, with 3 females and 3 males for each timepoint. (**c**) Schematic of the study design to test the target engagement for CK1δ/ε-specific PF-05236216 and CK1ε-specific PF-4,800,567 inhibitors in hTDP-43-ΔNLS mice. (**d**) Representative immunoblot images demonstrated reduced pTDP-43 levels in both soluble and insoluble protein fractions when mice were treated with either of the CK1 inhibitors at both doses tested. GAPDH signal was used to indicate the quality of fractionation between insoluble and soluble protein fractions. (**e**-**f**) Quantification of immunoblots showed pTDP-43 reduction in both soluble and insoluble protein fractions, with greater reduction observed in the soluble portion. Quantification of pTDP-43 reduction in soluble and insoluble protein fractions was computed as a normalized ratio between pTDP-43 and total TDP-43 signals. The signal intensity for each pTDP-43 band was first normalized to β-Actin, then calculated as a ratio relative to total TDP-43 levels (which was also normalized to β-Actin). Ratios were then compared to the untreated control group. This analysis incorporated normalization not only to an internal gene control within each sample using the housekeeping gene β-Actin, but also took into account the expression of the total TDP-43 (hTDP-43-ΔNLS transgene) since there may have been inherent genetic regulatory variability between samples. Two-way ANOVA, *p < 0.03, **p < 0.002, ***p < 0.0002, ****p < 0.0001. Error bars represent SEM

In our PK study, we sought to characterize the pharmacokinetic profiles for both CK1 inhibitors. We performed a single intraperitonially (IP) injection of the inhibitors into wild-type mice and collected whole brain tissue and plasma at multiple timepoints, which included 1-, 2-, 4-, 8-, and 24-hours post-injection (*n* = 6, 3 females and 3 males for each timepoint). The doses tested were 40 mg/kg for CK1δ/ε-specific PF-05236216 and 100 mg/kg for of CK1ε-specific PF-4,800,567, which were selected from our MTD study and published circadian studies utilizing these compounds or similar ones [51]. PK analysis in whole brain tissue revealed that the half-life for CK1δ/ε-specific PF-05236216 inhibitor was approximately 4–6 h (Fig. 4a), which was longer than the 1–2-hour half-life for CK1ε-specific PF-4,800,567 inhibitor (Fig. 4b). The longer half-life for compound CK1δ/ε-specific inhibitor (PF-05236216) was advanced into preclinical efficacy studies since it would allow us to have the temporal resolution to study CK1-dependent TDP-43 phosphorylation in vivo. Additional PK parameters can be found in Supplementary Table S1.

Next, we sought to demonstrate whether in vivo target engagement by the inhibitors could be achieved in hTDP-43-ΔNLS mice to establish a robust PD readout. Mice began treatment simultaneously as dox chow was removed to allow for transgene expression. Either CK1δ/ε-specific PF-05236216 inhibitor or CK1ε-specific PF-4,800,567 inhibitor was injected via IP at the pre-symptomatic stage once daily for 10 consecutive days at doses of 20 and 40 mg/kg and 50 and 100 mg/kg, respectively. The doses were selected based upon our MTD study and from referencing published circadian studies utilizing these compounds or similar ones [51]. The 10-day treatment length was selected based upon maximal pTDP-43 signal detection in immunoblots as previously mentioned (Fig. 2c). On the final 10th day, samples were collected 2–4 h after the last injection to account for the compound’s half-life (Fig. 4c) and processed for immunoblotting.

Generally, hTDP-43-ΔNLS mice treated for 10 days with CK1 inhibitor daily demonstrated PD effect via pTDP-43 (Fig. 4d). While pTDP-43 reduction was observed in mice treated with 100 mg/kg of CK1ε-specific PF-4,800,567 inhibitor, the effect was much more significant in mice treated with 20 mg/kg and 40 mg/kg of CK1δ/ε-specific PF-05236216 inhibitor, with 50% or more reduction observed in the soluble protein fraction (Fig. 4e). Notably, the reduced phosphorylation was more prominent in soluble protein fractions as opposed to insoluble protein fractions in hTDP-43-ΔNLS mice, suggesting the soluble pTDP-43 was more amenable to effects of the CK1 inhibitor (Fig. 4e-f), which was similar to results observed in our cellular models [26].

Altogether, these PK and PD data provided proof-of-concept for testing the long-term efficacy of CK1 inhibitors, particularly the CK1δ/ε-specific PF-05236216 inhibitor at a dosage of 40 mg/kg via IP injection, given its favorable profiles for longer PK half-life and effective PD target engagement of mechanism to reduce pTDP-43 levels in both soluble and insoluble protein fractions, and determine whether there would be a difference in TDP-43 proteinopathy, behavioral deficits, and other health metrics of hTDP-43-ΔNLS mice.

### Selective inhibition of CK1δ/ε reduces TDP-43 phosphorylation and Nf-L levels

**Fig. 5:**
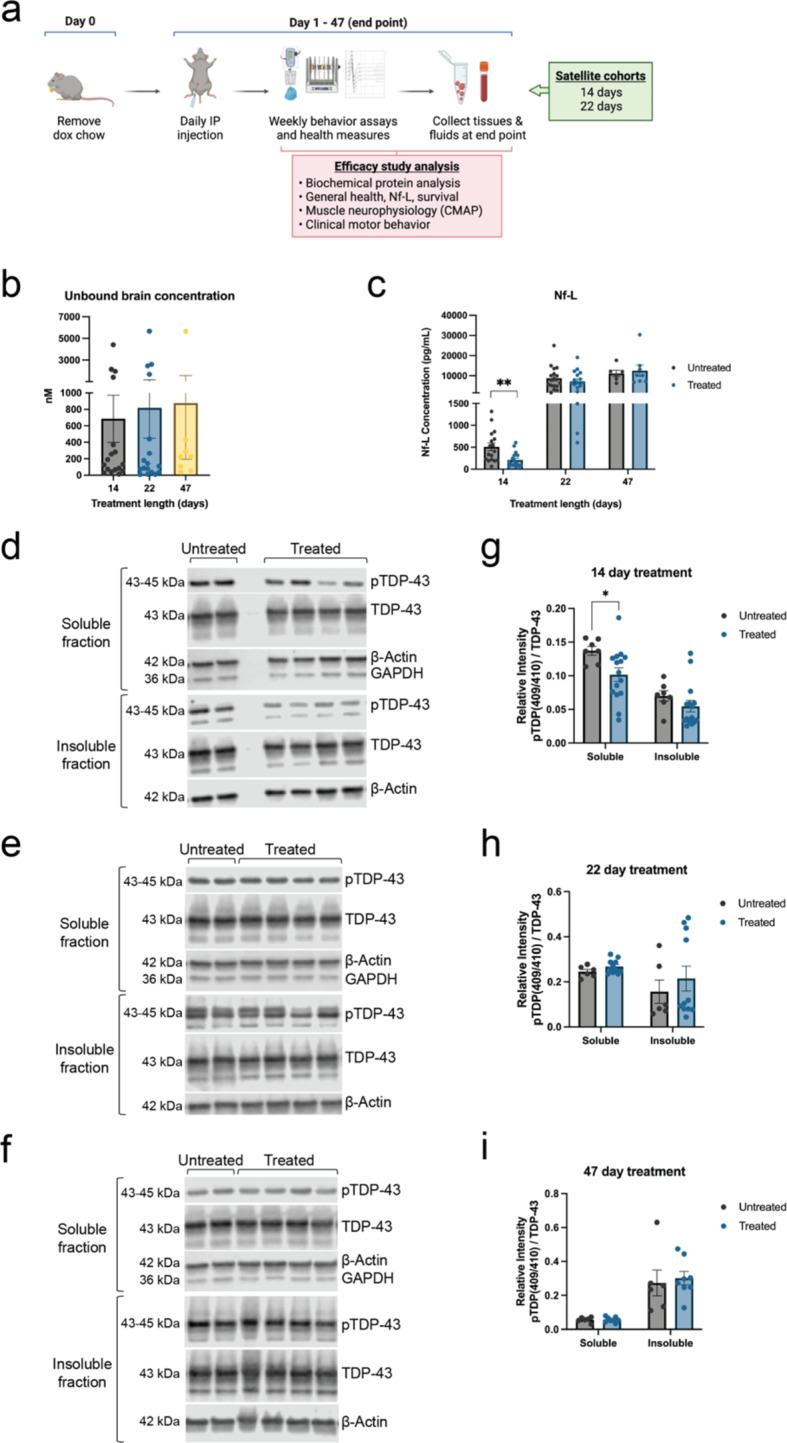
Mice treated with CK1δ/ε-selective inhibitor showed reduced TDP-43 phosphorylation and Nf-L levels. (**a**) Schematic of experimental design to test the efficacy of CK1δ/ε-specific inhibitor PF-05236216 in hTDP-43-ΔNLS mice. (**b**) Unbound inhibitor concentrations were detected and measured in the brain across 14, 22, and 47 days of inhibitor treatment, confirming the inhibitor could cross the blood brain barrier well. (**c**) Nf-L measurements from plasma samples across 14, 22, and 47 days of inhibitor treatment. Nf-L was significantly reduced by 2-fold in mice treated for 14 days. Mice treated for longer time frames did not show a difference compared to untreated mice. Mann-Whitney U test, *p < 0.03, **p < 0.002, ***p < 0.0002, ****p < 0.0001. Error bars represent SEM. (**d**) Representative immunoblot images demonstrated reduced pTDP-43 levels in both soluble and insoluble protein fractions when hTDP-43-ΔNLS mice were treated with CK1δ/ε-specific PF-05236216 inhibitor for 14 days. (**e**-**f**) 22-day and 47-day inhibitor treatment did not show a difference between treated and untreated groups. (**g**-**i**) Quantification of immunoblots showed greater pTDP-43 reduction in soluble protein fractions for 14-day long treated mice. No significant differences were detected in mice treated for either 22 or 47 days. Refer to Supplementary material file 2 for all immunoblots that were analyzed for this study. Quantification of pTDP-43 reduction in soluble and insoluble protein fractions was computed as a normalized ratio between pTDP-43 and total TDP-43 signals. The signal intensity for each pTDP-43 band was first normalized to β-Actin, then calculated as a ratio relative to total TDP-43 levels (which was also normalized to β-Actin). GAPDH signal was used to indicate the quality of fractionation between insoluble and soluble protein fractions. This analysis incorporated normalization not only to an internal gene control within each sample using the housekeeping gene β-Actin, but also took into account the expression of the total TDP-43 (hTDP-43-ΔNLS transgene) since there may have been inherent genetic regulatory variability between samples. Two-way ANOVA, *p < 0.03, **p < 0.002, ***p < 0.0002, ****p < 0.0001. Error bars represent SEM

With the inhibitor’s target engagement established, we then tested the efficacy of long-term treatment with CK1δ/ε-specific PF-05236216 inhibitor in hTDP-43-ΔNLS mice. Mice began treatment simultaneously as dox chow was removed to allow for transgene expression. The inhibitor was injected intraperitonially (IP) once daily at a dose of 40 mg/kg until mice reached either a natural humane endpoint or median survival, which was 47 days for the study (Fig. 5a). Mice were recorded and tested weekly for general health measures (body mass and survival), motor behavior assays (rotarod and forelimb grip strength tests), and muscle neurophysiology CMAP recordings. We also included in parallel, satellite cohorts for intermediary timepoint collections at days 14 and 22 throughout the treatment duration, which was determined based upon early-stage and late-stage observations in our *Csnk1e*-KO mouse study. The samples collected for biochemical assays included plasma for Nf-L measures, whole brain tissue and plasma for PK analysis of unbound inhibitor exposure levels, and frontotemporal cortex brain and spinal cord tissues for immunoblotting soluble and insoluble protein fractions (Fig. 5a).

In our study, we measured unbound inhibitor concentrations in the brain across 14, 22, and 47 days of treatment. Samples were collected 2–4 h after IP injection. We confirmed the 40 mg/kg dose of CK1δ/ε-specific inhibitor PF-05236216 had sufficient CNS penetration and distribution past the blood brain barrier, with some inhibitor availability in the brain tissues still remaining (Fig. 5b). The unbound inhibitor concentration in the brain was approximately 2-fold lower than the measurements from our PK study (Fig. 4a). This difference was likely due to the use of wild-type mice in the first study and our efficacy studies were from hTDP-43-ΔNLS mice. It can take approximately four to five half-lives for a compound to reach steady state when given at a regular interval. The measures for unbound inhibitor concentration in brains of mice treated for 14, 22, and 47 days likely represented steady state concentrations where a pharmacokinetic equilibrium would be reached, and the inhibitor concentrations would remain consistent throughout the duration of the efficacy study.

As previously stated, measuring Nf-L levels from plasma is emerging as a critical biomarker for neurodegenerative diseases [4, 5]. We observed a significant 2-fold reduction of Nf-L in mice treated for 14 days with CK1δ/ε-specific inhibitor PF-05236216 (Fig. 5c). This effect was not sustained with longer treatment durations of 22 or 47 days as treated mice had Nf-L levels similar to untreated control mice (Fig. 5c). The changes in Nf-L measurements strongly suggested that reducing TDP-43 phosphorylation via CK1 inhibition was sufficient to temporarily reduce neurodegeneration and delay disease progression, consistent with above studies.

Immunoblot analysis of brain protein lysates revealed that inhibitor treatment of 14 days resulted in decreased pTDP-43 levels in both soluble and insoluble protein fractions (Fig. 5d). Quantifying the immunoblots showed pTDP-43 reduction in soluble and insoluble protein fractions for 14-day treated mice (Fig. 5g). However, the effect was not sustained over longer periods of 22-day or 47-day inhibitor treatment (Fig. 5e-f), and no differences were detected between treated and untreated mice for 22-day or 47-day groups (Fig. 5h-i). We quantified the overall TDP-43 levels as well, which showed that it was not significantly altered in treated mice compared to untreated mice, providing confidence in the instances of pTDP-43 reduction (data not shown). The reduced pTDP-43 levels may correlate with the decreased Nf-L that was observed in 14-day treated mice, adding support to the suggestion that CK1δ/ε inhibitor treatment was sufficient to reduce neurodegeneration and delay disease progression in hTDP-43-ΔNLS mice. While targeting CK1δ/ε-mediated phosphorylation of TDP-43 demonstrated an effect with inhibitor treatment in the short term, it was not sustained with longer treatment periods.

### Selective inhibition of CK1δ/ε is insufficient to rescue motor deficits for functional benefit

**Fig. 6:**
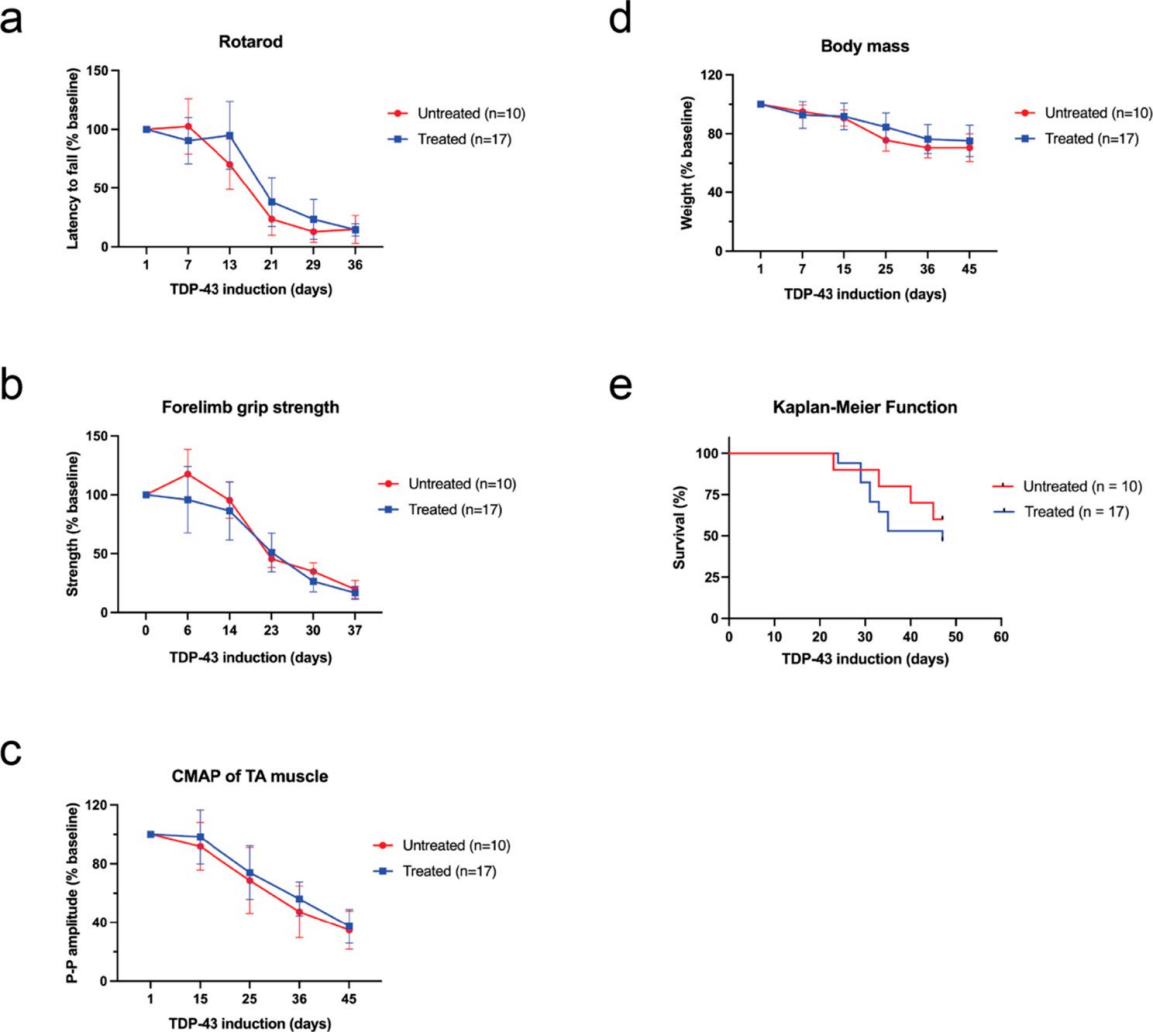
Treating mice with CK1δ/ε-selective inhibitor was insufficient to rescue motor deficits for functional benefit. (**a**) The cohort for rotarod, forelimb grip strength test, CMAP, body mass, and survival included 10 untreated hTDP-43-ΔNLS mice (5 male, 5 female) and 17 treated hTDP-43-ΔNLS mice (9 male, 8 female). Rotarod performance for treated mice did not improve as compared to untreated control mice. (**b**) Forelimb grip strength test also did not display improvements. (**c**) Treated mice did not display changes in CMAP recordings of the TA muscles. (**d**) The overall health of mice treated with inhibitor was not exacerbated since the body mass declined similarly as untreated mice. (**e**) Treated mice reached median survival and end stage faster than untreated mice, likely due to an unintended adverse event with long-term treatment with this particular CK1δ/ε-selective inhibitor. Two-way ANOVA with repeated measures and Dunnett’s multiple comparisons, *p < 0.03, **p < 0.002, ***p < 0.0002, ****p < 0.0001. Error bars represent SEM

We conducted motor behavior assays on rotarod and forelimb grip strength tests, recorded CMAPs of the TA muscle, and noted general health measures for body mass and survival. For the rotarod assay, forelimb grip strength test, and neurophysiology CMAP recordings, there was no significant difference throughout disease progression over the course of 47 days between treated and untreated groups (Fig. 6a-c). Body mass data suggested that while there was no difference between groups, the symptoms of the treated mice were not exacerbated compared to untreated mice which indicated a safe metabolic profile for the inhibitor (Fig. 6d).

The Kaplan-Meier curve showed that treated mice reached median survival and end stage before untreated mice did (Fig. 6e). We were unable to collect samples from mice found at end stage due to long and undetermined postmortem interval (PMI) that would have compromised sample quality. During our dose-finding and PK/PD studies, we identified the MTD for CK1δ/ε-specific PF-05236216 inhibitor was 80 mg/kg, which resulted in fatal neurological side effects within 24 h of a single IP injection. This indicated that there may also be long-term side effects of CK1δ/ε-specific PF-05236216 inhibitor treatment even when used at a dose below the MTD.

We suspected there may have been other kinases or altered genes that contributed to the survival outcomes of treated mice. To investigate this, we pursued global transcriptomic studies with bulk-RNA sequencing of samples from the 14-day and 22-day treated and untreated mice groups, noting that expression levels of kinases do not necessarily equate with kinase activity (Supplementary figure S3a). There remain technical challenges in isolating motor neurons for cell-specific transcriptome profiling, thus, we explored bulk-RNA sequencing as an initial screen. Comparing highly differentiated genes between the two timepoints revealed *MAP3K6* as significantly upregulated in the treated groups compared to untreated groups (Supplementary figure S3b). *MAP3K6* is a serine/threonine kinase that regulates vascular endothelial growth factor (VEGF) expression and angiogenesis for the formation of new blood vessels, and also activates the JNK signaling pathway which regulates programmed cell death and apoptosis [46, 49]. The significant upregulation of *MAP3K6* expression may have imposed some neurotoxicity and likely contributed to the premature death of treated mice and would be considered an unintended adverse event with long-term CK1δ/ε-specific PF-05236216 inhibitor treatment. To ascertain whether the inhibitor treatment was significantly detrimental to the health of the mice, we performed gene set enrichment analysis (GSEA) between the 22-day treated, 22-day untreated, and non-Tg WT mice and did not identify any notable differences between the gene ontology (GO) groups and enrichment scores (Supplementary figure S3c-d).

We had concerns that our IP daily dosing delivery strategy may not have allowed for sustained inhibition of CK1δ/ε-mediated phosphorylation for TDP-43 throughout the day, so we conducted an additional efficacy study using special diet chow formulated with CK1δ/ε-specific PF-05236216 inhibitor for continuous delivery of inhibitor. We tested a lower dose of 20 mg/kg due to the potential long-term side-effects uncovered from our transcriptomic profiling data. Ultimately, whether mice were treated with IP injection or continuous chow infusion, the results were comparable on pTDP-43 levels in immunoblots and there was no significant improvement for motor deficits or other health measures (Supplementary figure S4a-e).

## Discussion

In this study, we advanced our previous findings from in vitro and cellular experimental systems to the in vivo hTDP-43-ΔNLS mouse model of ALS and related TDP-43 proteinopathies. Overall, we found that targeting CK1-mediated phosphorylation via genetic *Csnk1e* knockout and CK1δ/ε- selective PF-05236216 chemical inhibition in hTDP-43-ΔNLS mice delayed the formation of pTDP-43, lowered Nf-L levels, and temporarily shifted the survival curve favorably, but overall did not rescue neurodegeneration to a significant level that ameliorated disease phenotypes or for functional benefit. The positive aspects of our results support the disease-promoting role of TDP-43 phosphorylation in ALS, underscoring the aggressiveness of the mouse model and the complexity of TDP-43 phosphorylation. Selective CK1δ/ε inhibition showed moderate beneficial effects in vivo, but other kinases seem to be involved. Despite the uncertain value of TDP-43 phosphorylation as a target for therapy, pTDP-43 could still be a valuable marker for efficacy of therapeutics that aim to modify TDP-43 aggregation.

A limitation of our *Csnk1e* genetic deletion study and important consideration when interpreting these results was that the presence and functional contribution of CK1δ still remained in the experimental system. We had thoroughly characterized the *Csnk1e-*KO mice for *Csnk1d* expression, which indicated no significant alterations when *Csnk1e* was removed. Despite the lack of change in expression, CK1δ may have increased kinase activity functionally and possibly compensated for the lack of CK1ε in vivo. Ideally, an in vivo kinase assay would be used to determine if CK1δ activity was increased but identifying and developing the assay for a CK1δ-specific sensitive biomarker as a readout is challenging. For this, we also attempted immunoblotting, global proteomic mass spectrometry profiling, and phospho-proteomic approaches to see if we could detect increased phosphorylation of downstream CK1δ targets (i.e. PER1, PER2, etc.), but the antibody tools were inadequate and mass spectrometry protein phosphorylation detection lacked sensitivity and was highly variable. Thus, we leveraged pharmacologic strategies with CK1 kinase inhibitors to study the simultaneous contribution of both CK1δ- and CK1ε-mediated phosphorylation of TDP-43.

Our work reinforces and extends the results of another study that tested CK1δ-selective inhibitor (IGS-2.7) in the TDP-43 (A315T) mutation mouse model [34]. Our studies included comprehensive measures of not only biochemical and histological readouts of TDP-43 phosphorylation, but also behavior analysis and functional assays. Despite differences in the transgenic mouse model and inhibitor used, the study testing IGS-2.7 in TDP-43 (A315T) mice concluded that inhibitor treatment preserved spinal cord motor neurons and reduced glial immunoreactivity [34], which aligns with our findings on the disease-promoting role of TDP-43 phosphorylation in ALS. Essentially, we were able to thoroughly demonstrate that CK1δ/ε inhibition delayed the formation of pTDP-43, lowered Nf-L levels, and temporarily shifted the survival curve favorably, thereby reducing TDP-43 toxicity in vivo.

Throughout our studies, we examined the effect of CK1δ/ε inhibitor treatment on pTDP-43 formation in both soluble and insoluble protein fractions at multiple timepoints. Not all studies of TDP-43 phosphorylation in ALS in vitro cellular and in vivo mouse models have examined multiple timepoints and in both protein fractions, which we have demonstrated is a critical component for insight into the chronological dynamics of neurodegenerative disease mechanisms and progression. Our studies in *Csnk1e-*KO mice showed that phosphorylation did not contribute to the formation of insoluble TDP-43 and likely affected the liquid-liquid phase separation properties and aggregation propensity of TDP-43 by disrupting its self-self-interaction of the C-terminal low-complexity glycine-rich domain. Studies have suggested that phosphorylation of TDP-43 occurred after aggregate formation and that the modification was not a prerequisite for aggregation [11, 21]. The pTDP-43 reduction was more pronounced in the soluble fraction compared to insoluble fraction, indicating that the soluble TDP-43 may have been more amenable to the inhibitor treatment. In addition, cryo-EM studies of TDP-43 structure revealed that S410 is exposed on the surface while S409 is buried in the interior of the structure, indicating that there may be variable effectiveness at targeting S409/410 sites, especially as TDP-43 self-aggregates into an insoluble state [29].

Our studies of *Csnk1e* gene deletion knockout and CK1δ/ε inhibitor treatment in this specific hTDP-43-ΔNLS mouse model suggest there may have been a critical period early in disease progression, prior to any motor deficit symptom onset, where CK1-mediated phosphorylation actively affected TDP-43 solubility, but then had a decreased and more limited role at later stages of disease as the hTDP-43-ΔNLS overexpression dominated in these mice. Alternatively, inhibiting CK1δ/ε-mediated phosphorylation could have induced alternative compensatory routes of TDP-43 phosphorylation. An open question that remains is how TDP-43 degradation may occur in ALS disease, and whether phosphorylation affects the degradation rate of TDP-43. Taken together, phosphorylation may act as a mechanistic switch for TDP-43 cytoplasmic accumulation and either prevent the initial formation of TDP-43 aggregates or alter the existing insoluble TDP-43 aggregates into soluble TDP-43. Nevertheless, our work adds to a growing body of literature underpinning the role of phosphorylation in mediating TDP-43 solubility and aggregation.

There are a variety of ALS mouse models of TDP-43 proteinopathies, each with their own set of limitations [32, 42]. We selected the inducible hTDP-43-ΔNLS model for our studies due to its robust phenotypes of cytoplasmic TDP-43 accumulation with detectable phosphorylation and motor behavior deficits and inducibility, which were lacking in other ALS mouse models. However, this model did not reflect the focal onset and spread of TDP-43 proteinopathy across different regions over time, as seen in ALS patients. The hTDP-43-ΔNLS transgene remained highly expressed throughout the disease progression [50], which may have been too aggressive and fast-progressing for our investigation. TDP-43 is known to self-aggregate, so it is plausible for TDP-43 overexpression beyond a saturation point to become a consequence of the model itself. Compared to human ALS disease, which is related to intrinsic changes of TDP-43 protein and not protein overexpression, this limits the translatability of our results. Perhaps titrating the dox dose and maintaining the mice on a low dose of dox chow throughout the study would have helped to temper the transgene expression levels so mice would mildly decline while still generating the TDP-43 proteinopathy phenotypes of interest.

The hTDP-43-ΔNLS transgene being overexpressed was a mutated TDP-43 that contained a defective nuclear localization signal (NLS), so TDP-43 could not translocate back into the nucleus and was retained and accumulated in the cytoplasm of cells. Thus, we were unable to study the effect of CK1-phosphorylation on the nucleo-cytoplasmic shuttling of TDP-43 since it has been suggested that CK1δ inhibition could ensure the physiological nuclear compartmentalization of TDP-43 and regulated the nucleo-cytosol translocation of TDP-43 [3]. A study performed in AD lymphoblasts found that a CK1δ/ε inhibitor restored TDP-43 homeostasis and prevented cell-to-cell propagation of TDP-43 pathology [33]. Such a study would provide insight on the relative contributions of TDP-43 nuclear loss-of-function and cytoplasmic gain-of-toxicity in driving ALS pathogenesis as well as the role of CK1-mediated phosphorylation in potentially regulating these disease mechanisms. There may be two independent roles for TDP-43 phosphorylation during disease depending on its cellular localization in the nucleus versus the cytoplasm.

Interestingly, a study on the FUS mutation-associated ALS (where *FUS* mutation-associated ALS patients do not develop TDP-43 proteinopathy) found that CK1δ/ε-mediated phosphorylation of FUS protein increased the solubility of FUS in vitro and overexpression of CK1δ significantly rescued degeneration in a transgenic fly model [25]. However, for ALS cases with TDP-43 proteinopathy, phosphorylation may have an alternative effect since we demonstrated that CK1δ/ε-selective inhibition could reduce Nf-L levels and moderately rescue neurodegeneration in mice. Given that CK1δ/ε-selective inhibition alone was not sufficient to ameliorate disease progression for functional benefit, a strategy targeting an alternative priming kinase, or multiple kinases may yield a more significant rescue effect (discussed in [26]).

In summary, the role of phosphorylation to TDP-43 in ALS and TDP-43 proteinopathies is complex and nuanced. Phosphorylation likely participates in neurotoxicity but may not be a sole driver of disease toxicity. Altogether, these results suggest that CK1 inhibitors may have potential as an ALS therapy in combination with other drug targeting TDP-43 pathology.

## Conclusions

In our study, we found that targeting CK1-mediated phosphorylation using two different strategies, via genetic *Csnk1e* knockout or CK1δ/ε- selective PF-05236216 chemical inhibition, in hTDP-43-ΔNLS mice delayed the formation of pTDP-43, lowered Nf-L levels, and temporarily shifted the survival curve favorably. This targeting was not sufficient or sustained to fully eliminate toxicity or ameliorate disease phenotypes for functional benefit, recognizing that it was tested in an aggressive mouse model. Our results suggest that manipulation of TDP-43 phosphorylation by selective CK1δ/ε inhibition may be better suited as a combination or adjunctive drug therapy with other TDP-43-pathology targeting drugs.

## Electronic supplementary material

Below is the link to the electronic supplementary material.

Supplementary Material 1

Supplementary Material 2

Supplementary Material 3

Supplementary Material 4

Supplementary Material 5

Supplementary Material 6

## Data Availability

No datasets were generated or analysed during the current study.

## Abbreviations

ALS: Amyotrophic lateral sclerosis
CMAP: Compound muscle action potential
CNS: Central nervous system
CSNK1D: Casein kinase 1 delta gene
CSNK1E: Casein kinase 1 epsilon gene
CK1δ: Casein kinase 1 delta protein
CK1ε: Casein kinase 1 epsilon protein
dox: Doxycycline
GSEA: Gene set enrichment analysis
GO: Gene ontology
IP: Intraperitoneal
MTD: Maximal tolerated dose
Nf-L: Neurofilament-Light
NLS: Nuclear localization signal
PD: Pharmacodynamic
PK: Pharmacokinetic
pTDP-43: Phosphorylated TDP-43
S409/410: Serine 409/410 phosphorylation sites on TDP-43
SEM: Standard error of the mean
TA: Tibialis anterior
TDP-43: TAR DNA-binding protein 43
