# Supplementary figures and images for: CK1δ/ε-mediated TDP-43 phosphorylation contributes to early motor neuron disease toxicity in amyotrophic lateral sclerosis
Source: Acta Neuropathol Commun. 2024 Dec 4;12:187. doi: 10.1186/s40478-024-01902-z (PMC11619411; doi:10.1186/s40478-024-01902-z)

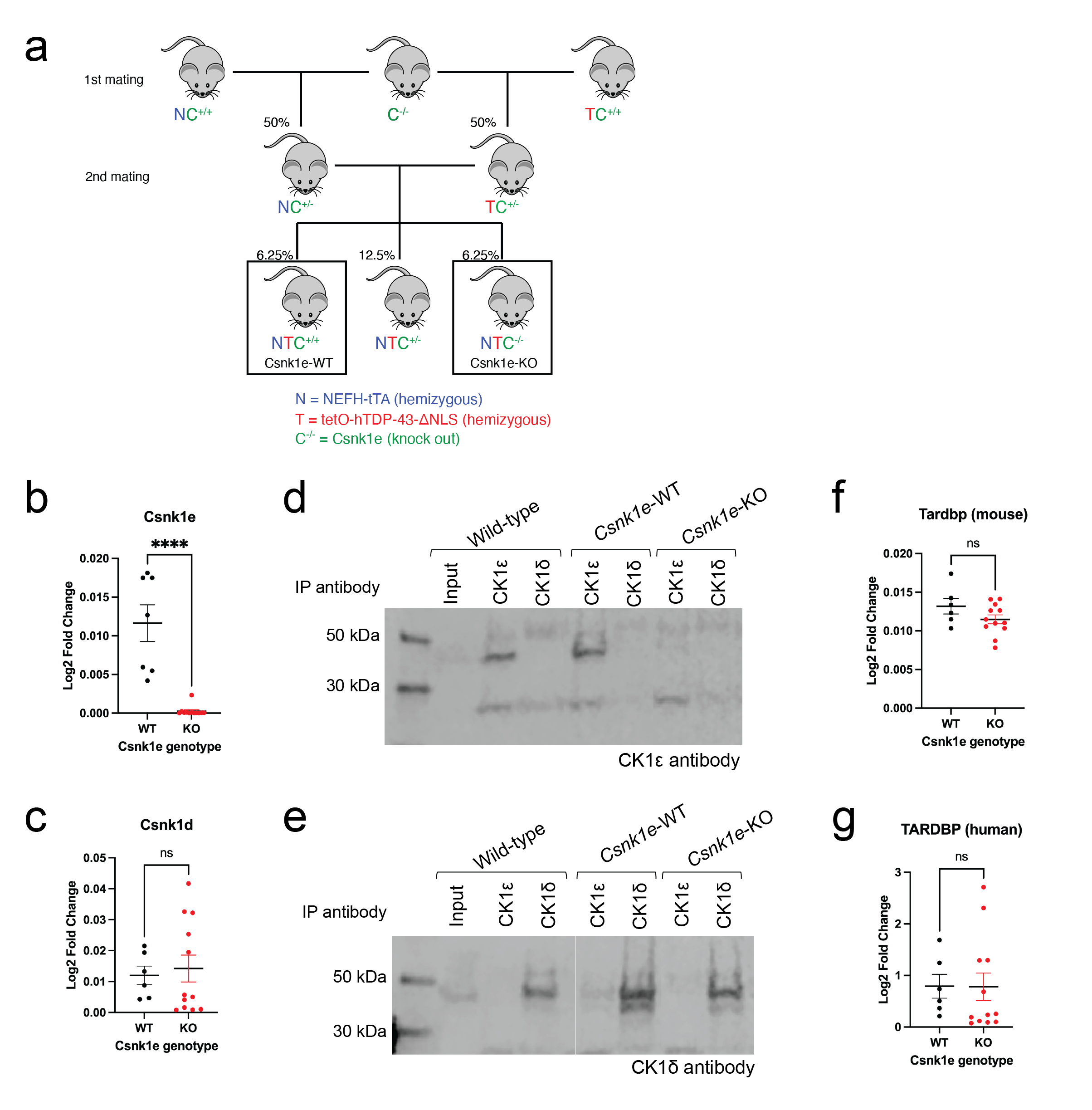

Supplement: Supplementary file 1 — Supplementary Material 1 [file 40478_2024_1902_MOESM1_ESM.png]

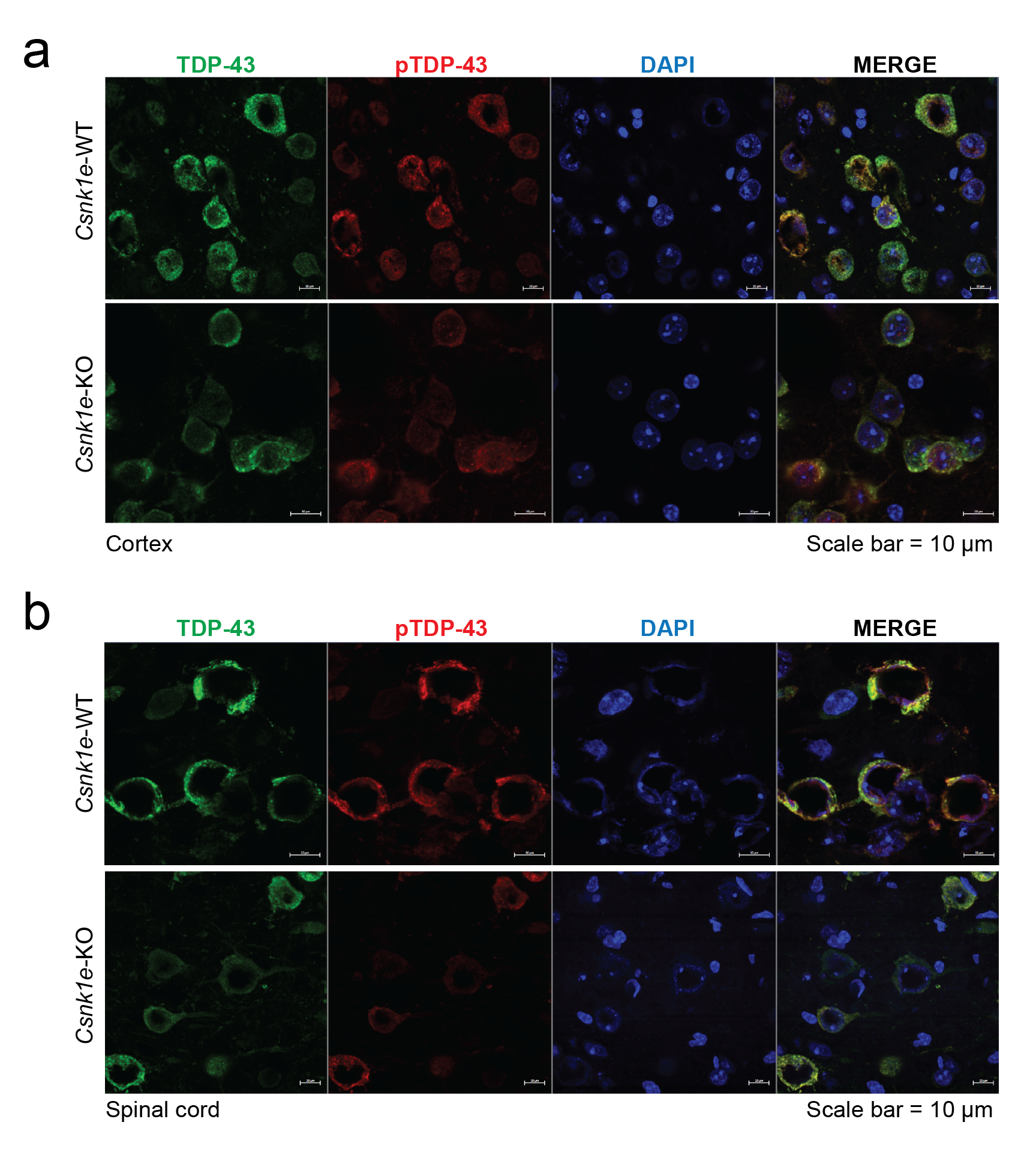

Supplement: Supplementary file 2 — Supplementary Material 2 [file 40478_2024_1902_MOESM2_ESM.png]

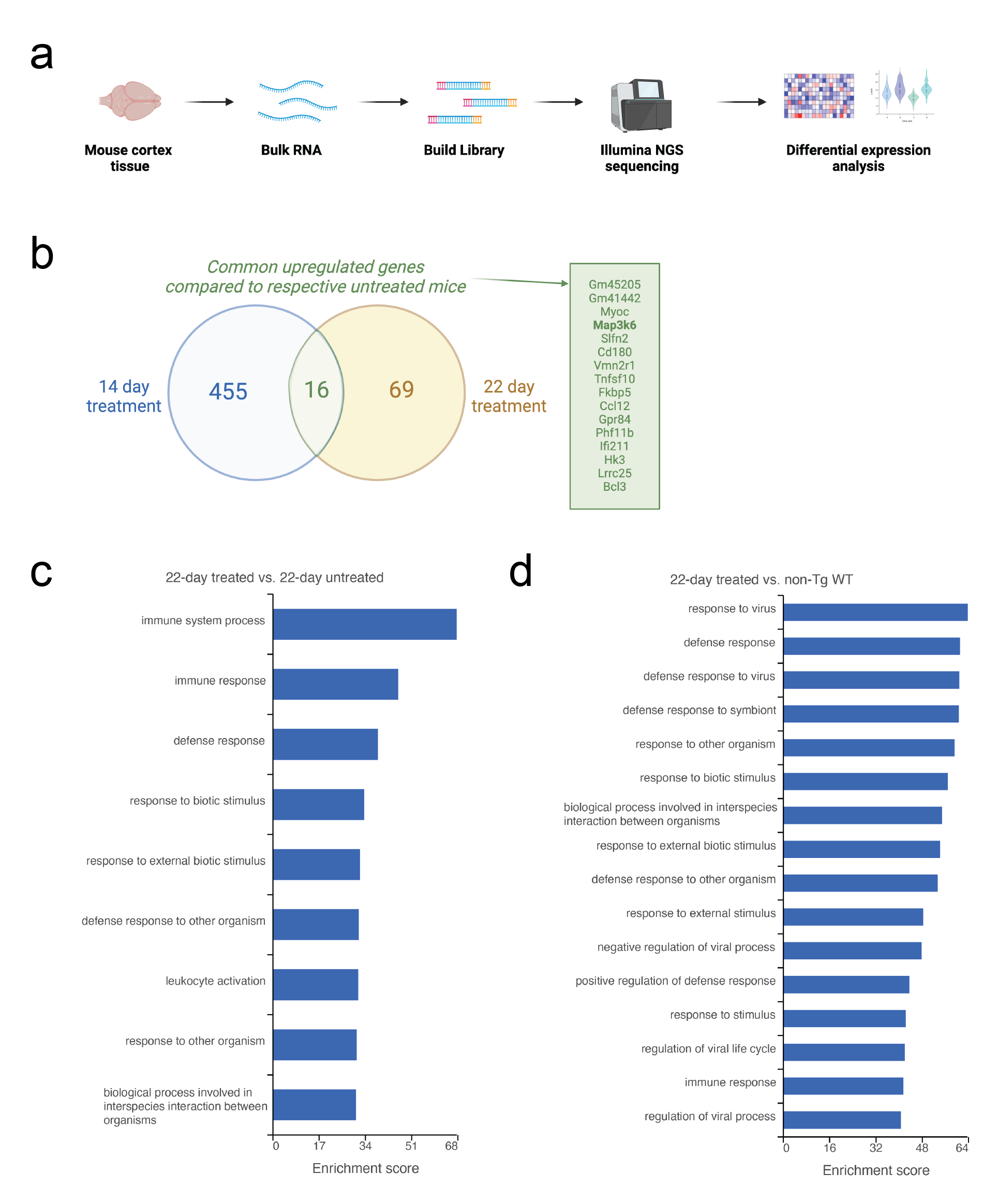

Supplement: Supplementary file 3 — Supplementary Material 3 [file 40478_2024_1902_MOESM3_ESM.png]

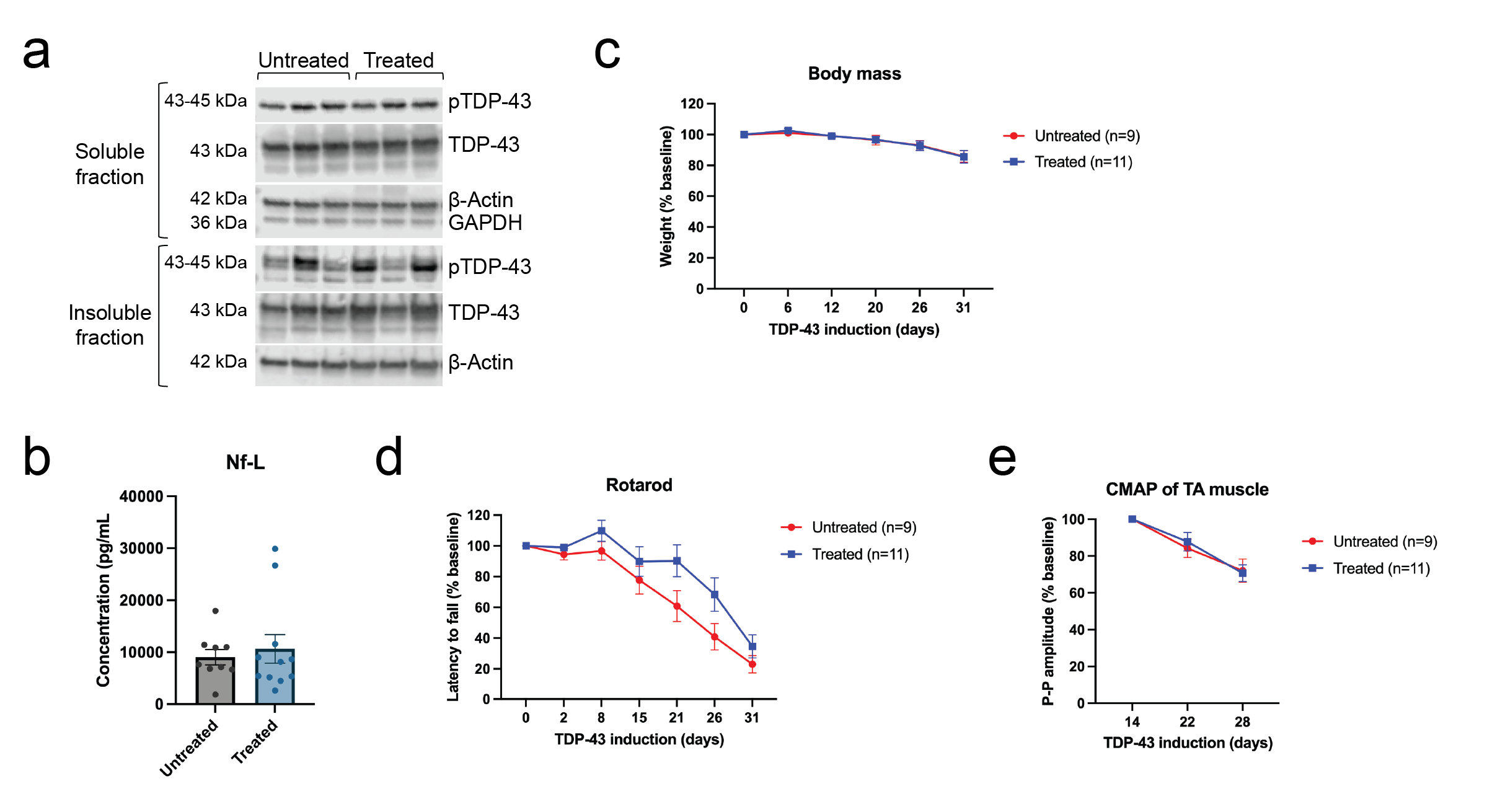

Supplement: Supplementary file 4 — Supplementary Material 4 [file 40478_2024_1902_MOESM4_ESM.png]
