# Supplementary material for: CK1δ/ε-mediated TDP-43 phosphorylation contributes to early motor neuron disease toxicity in amyotrophic lateral sclerosis
Source: Acta Neuropathol Commun. 2024 Dec 4;12:187. doi: 10.1186/s40478-024-01902-z (PMC11619411; doi:10.1186/s40478-024-01902-z)

**Supplemental Information File 2.**

Raw blot of soluble pTDP-43 (S409/410) (Samples lanes 3-12, top) and insoluble pTDP-43 (S409/410) (Samples lanes 3-12, bottom) shown in Fig 2C.


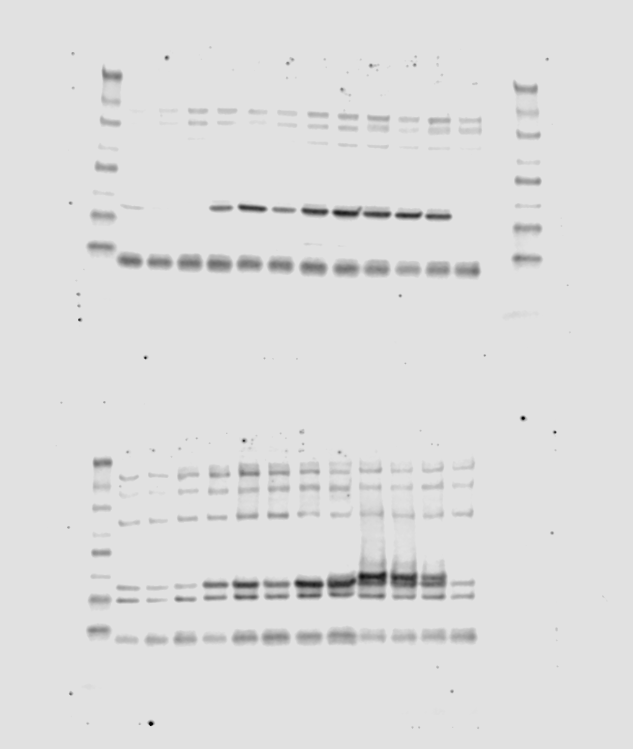


Raw blot of soluble TDP-43 (Samples lanes 3-12, top) and insoluble TDP-43 (Samples lanes 3-12, bottom) shown in Fig 2C.


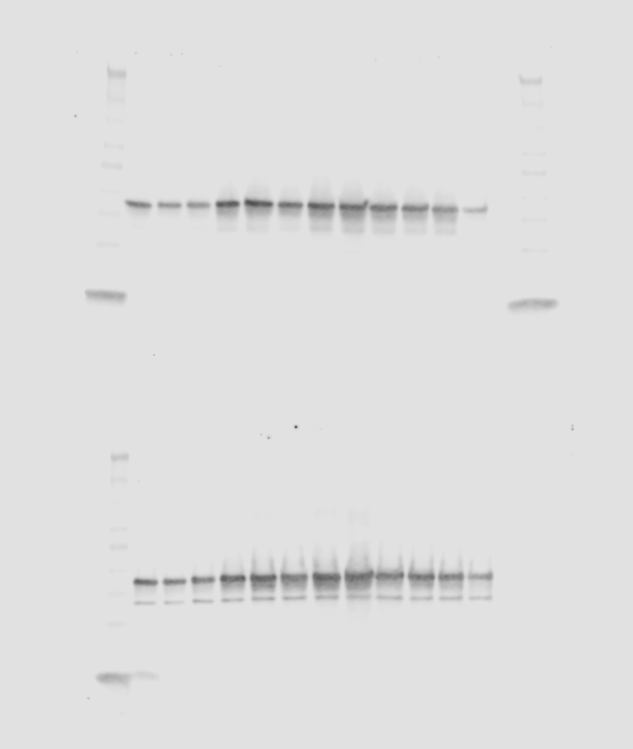


Raw blot of soluble β-actin (Samples lanes 3-12, top) and insoluble β-actin (Samples lanes 3-12, bottom) shown in Fig 2C.


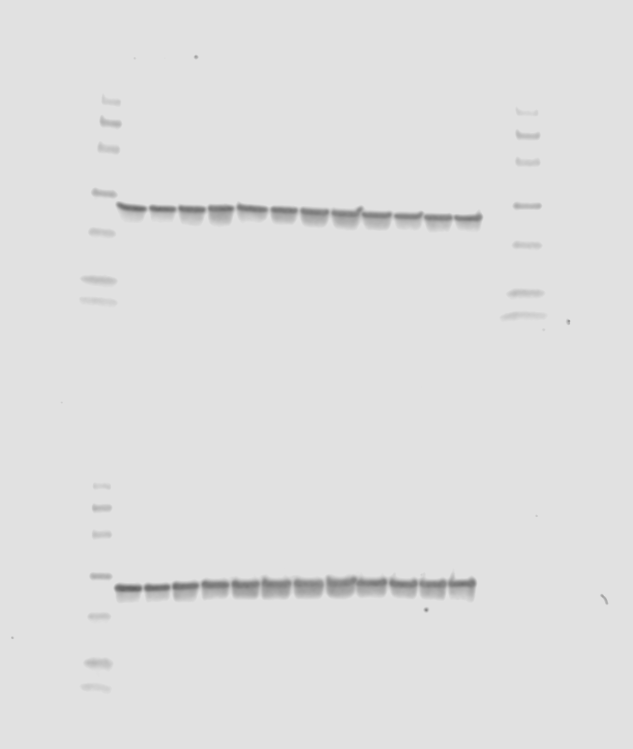


Raw blot of soluble pTDP-43 (S409/410) (sample lanes 2-8) and insoluble pTDP-43 (S409/410) (samples lanes 9-15) shown in Fig 3D.


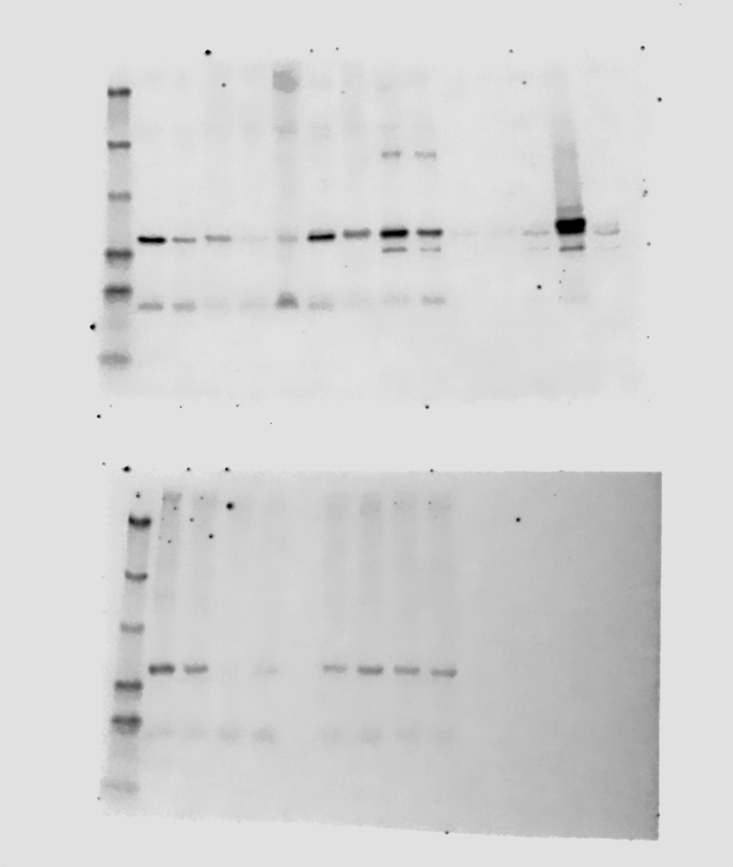


Raw blot of soluble TDP-43 (sample lanes 2-8) and insoluble TDP-43 (samples lanes 9-15) shown in Fig 3D.


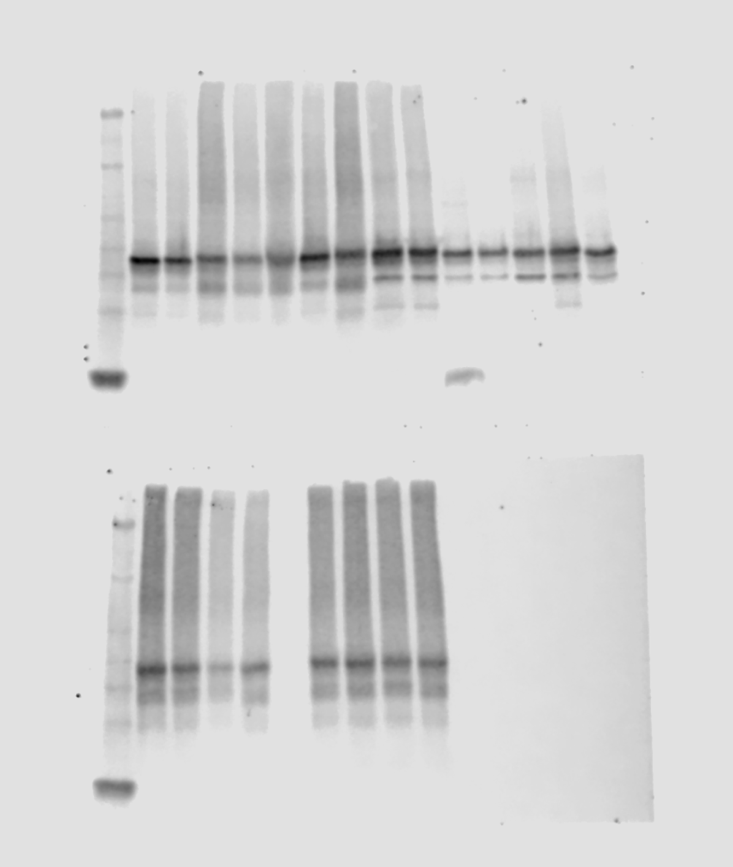


Raw blot of soluble β-actin and GAPDH (sample lanes 2-8) and insoluble β-actin and GAPDH (samples lanes 9-15) shown in Fig 3D.


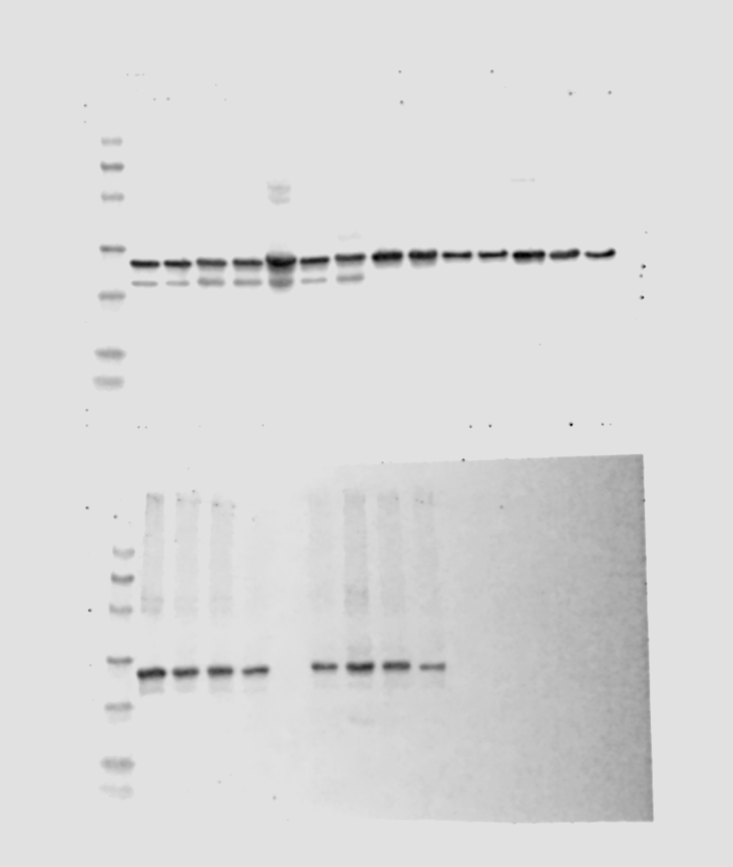


Raw blot of untreated and PF-05236216 treated mice. Soluble pTDP-43 (S409/410) (sample lanes 2-6, 13-15) shown in Fig 4D.


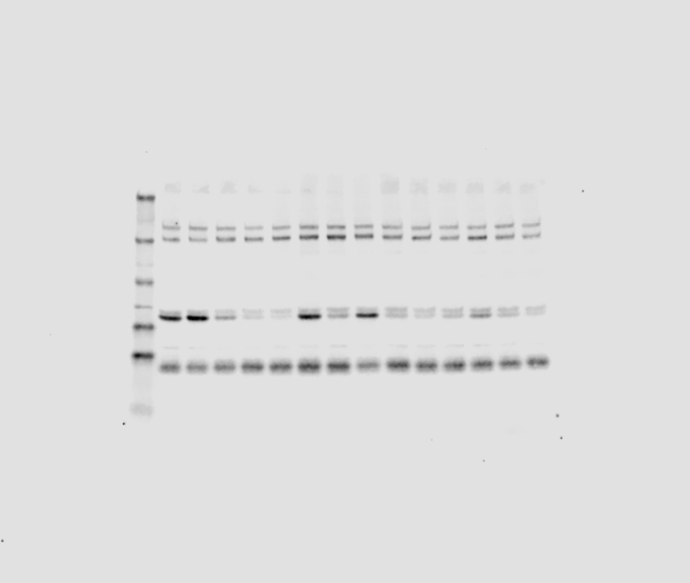


Raw blot of untreated and PF-05236216 treated mice. Insoluble pTDP-43 (S409/410) (sample lanes 2-6, 13-15) shown in Fig 4D.


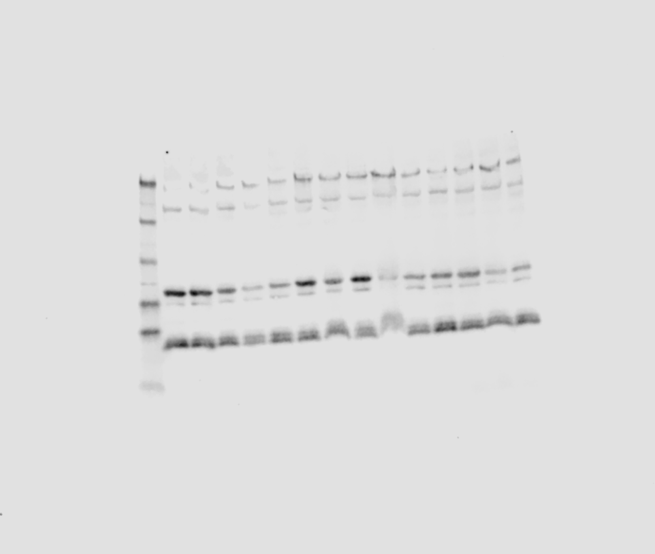


Raw blot of untreated and PF-05236216 treated mice. Soluble TDP-43 (sample lanes 2-6, 13-15) shown in Fig 4D.


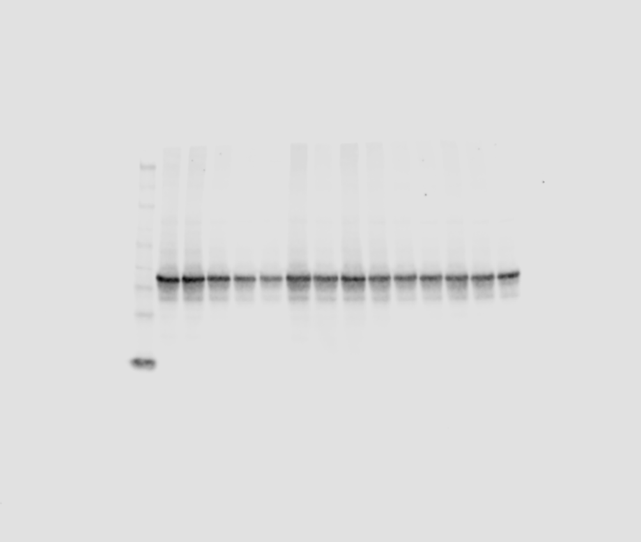


Raw blot of untreated and PF-05236216 treated mice. Insoluble TDP-43 (sample lanes 2-6, 13-15) shown in Fig 4D.


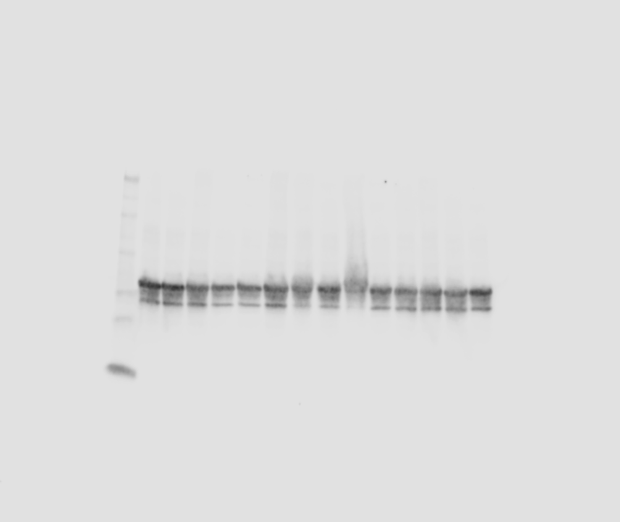


Raw blot of untreated and PF-05236216 treated mice. Soluble β-actin and GAPDH (sample lanes 2-6, 13-15) shown in Fig 4D.


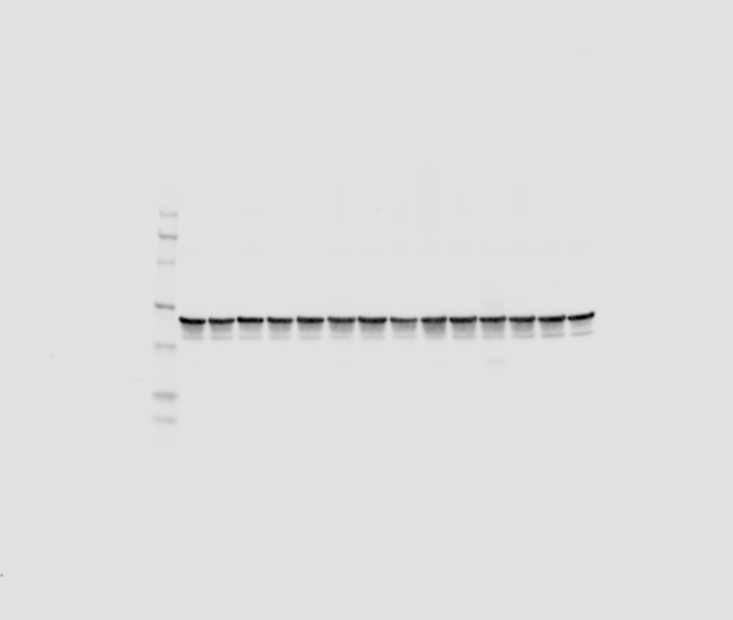


Raw blot of untreated and PF-05236216 treated mice. Insoluble β-actin and GAPDH (sample lanes 2-6, 13-15) shown in Fig 4D.


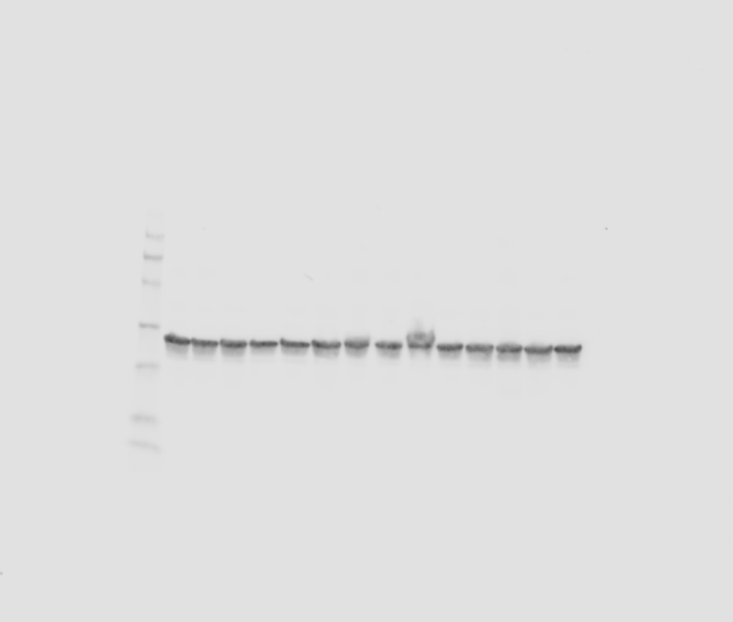


Raw blot of PF-4800567 treated mice. Soluble pTDP-43 (S409/410) (sample lanes 2-7) and insoluble pTDP-43 (S409/410) (samples lanes 10-15) shown in Fig 4D.


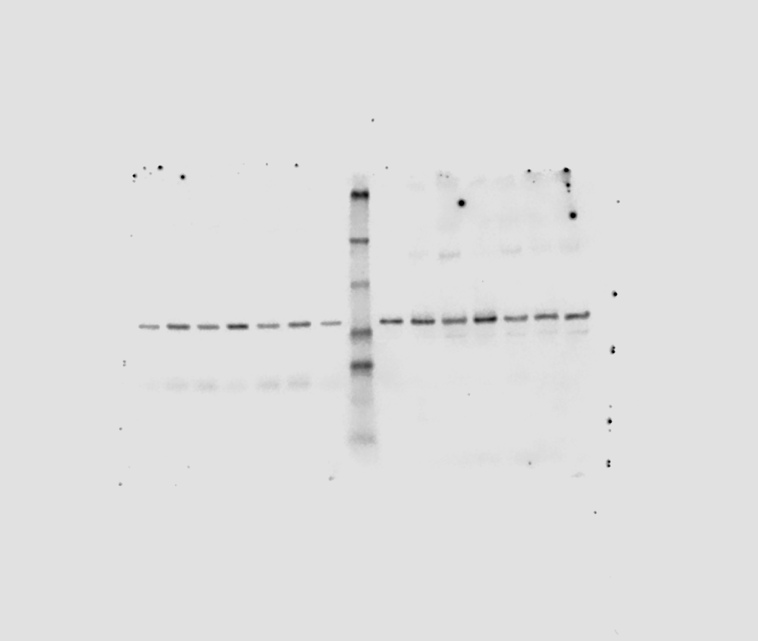


Raw blot of PF-4800567 treated mice. Soluble TDP-43 (sample lanes 2-7) and insoluble TDP-43 (samples lanes 10-15) shown in Fig 4D.


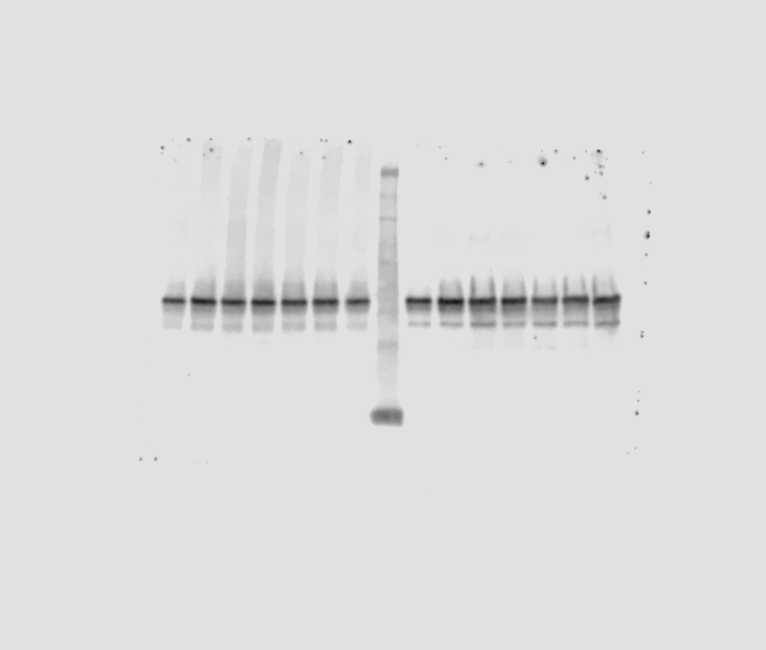


Raw blot of PF-4800567 treated mice. Soluble β-actin and GAPDH (sample lanes 2-7) and insoluble β-actin and GAPDH (samples lanes 10-15) shown in Fig 4D.


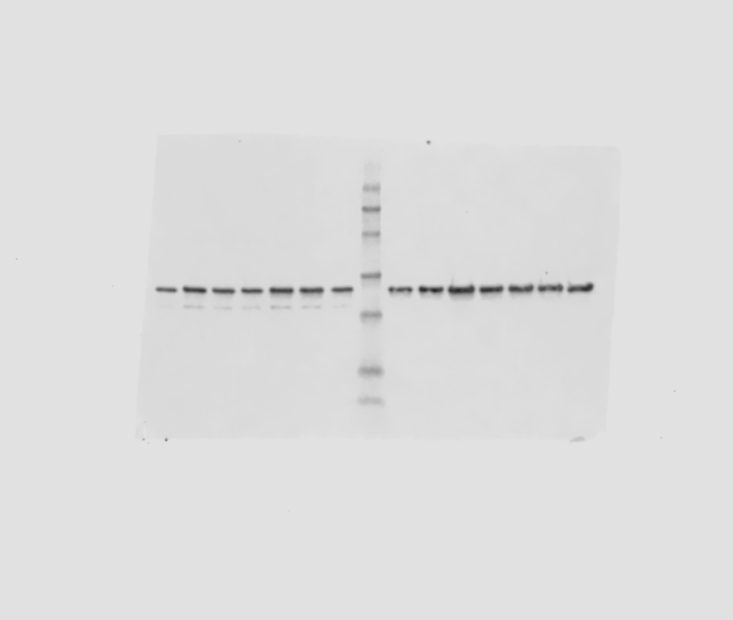


Raw blot of soluble pTDP-43 (S409/410) (sample lanes 4-10, top) and insoluble pTDP-43 (S409/410) (samples lanes 4-10, bottom) shown in Fig 5D and used for quantification in Fig 5G.


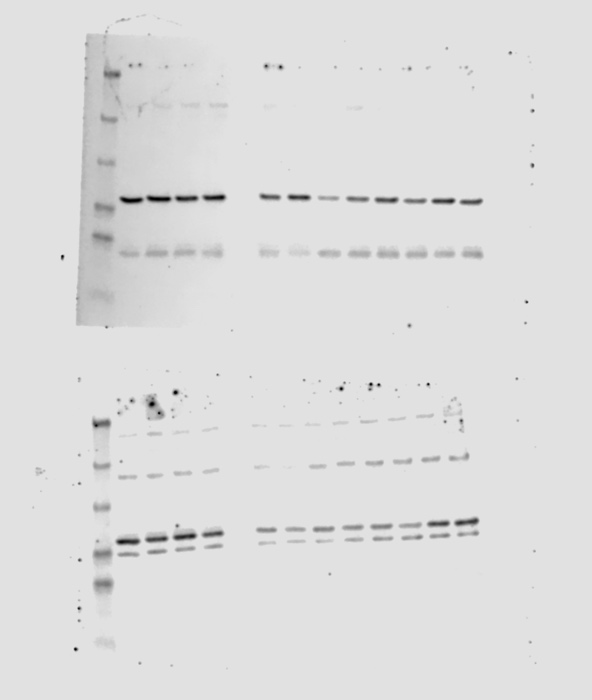


Raw blot of soluble TDP-43 (sample lanes 4-10, top) and insoluble TDP-43 (samples lanes 4-10, bottom) shown in Fig 5D and used for quantification in Fig 5G.


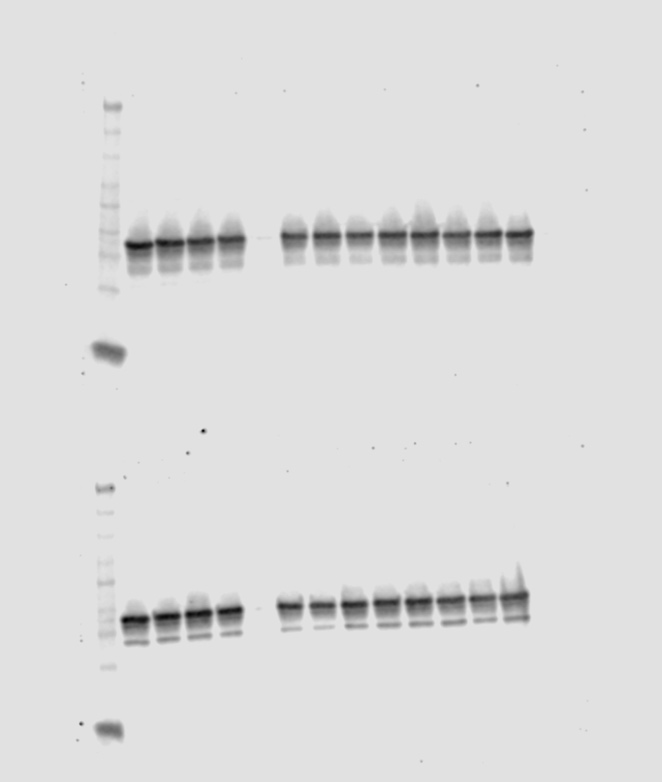


Raw blot of soluble β-actin and GAPDH (sample lanes 4-10, top) and insoluble β-actin and GAPDH (samples lanes 4-10, bottom) shown in Fig 5D and used for quantification in Fig 5G.


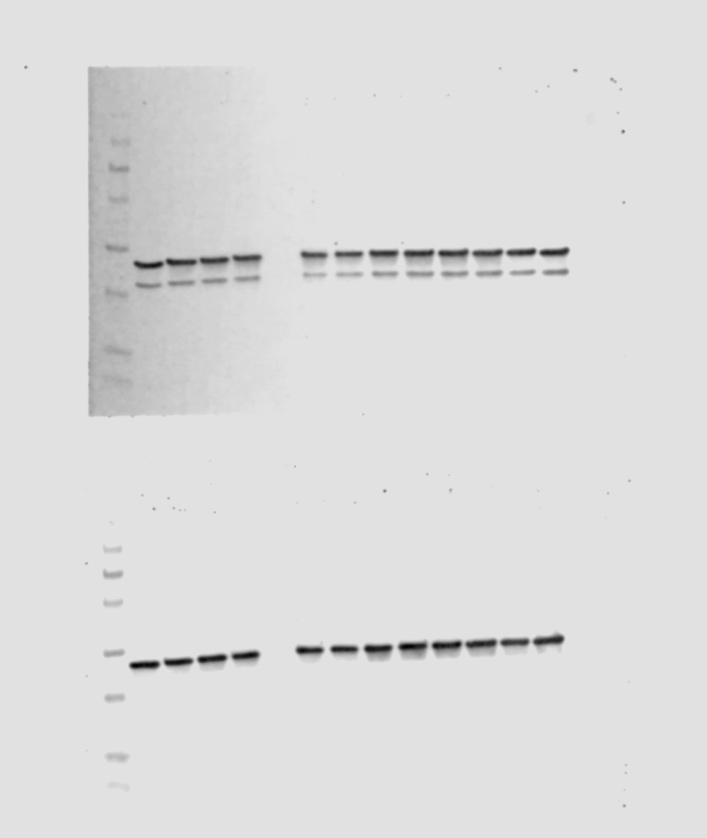


Additional raw blot of soluble pTDP-43 (S409/410) (top) and insoluble pTDP-43 (S409/410) (bottom) used for quantification in Fig 5G.


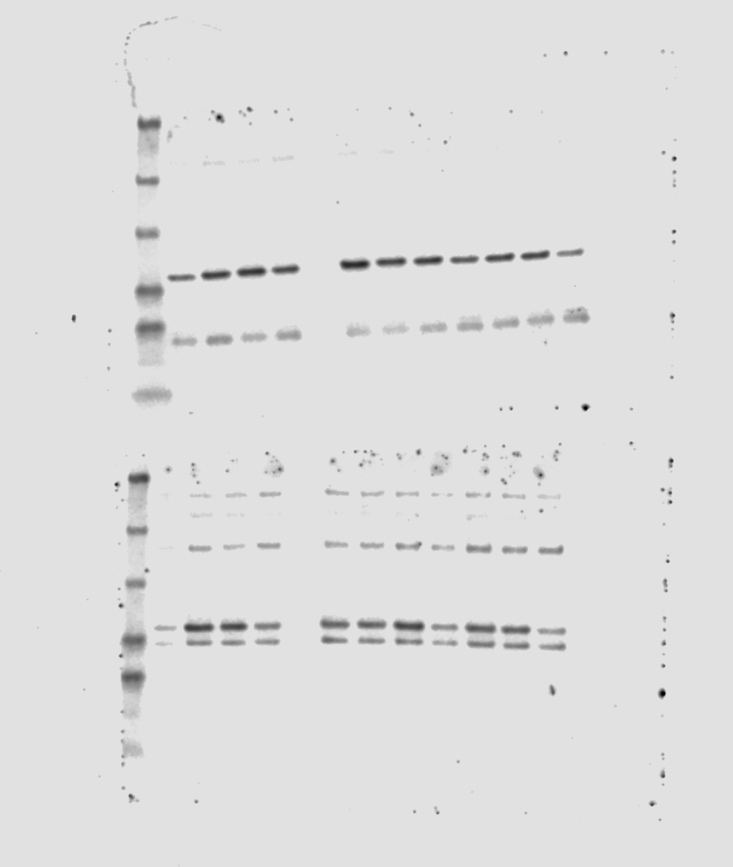


Additional raw blot of soluble TDP-43 (top) and insoluble TDP-43 (bottom) used for quantification in Fig 5G.


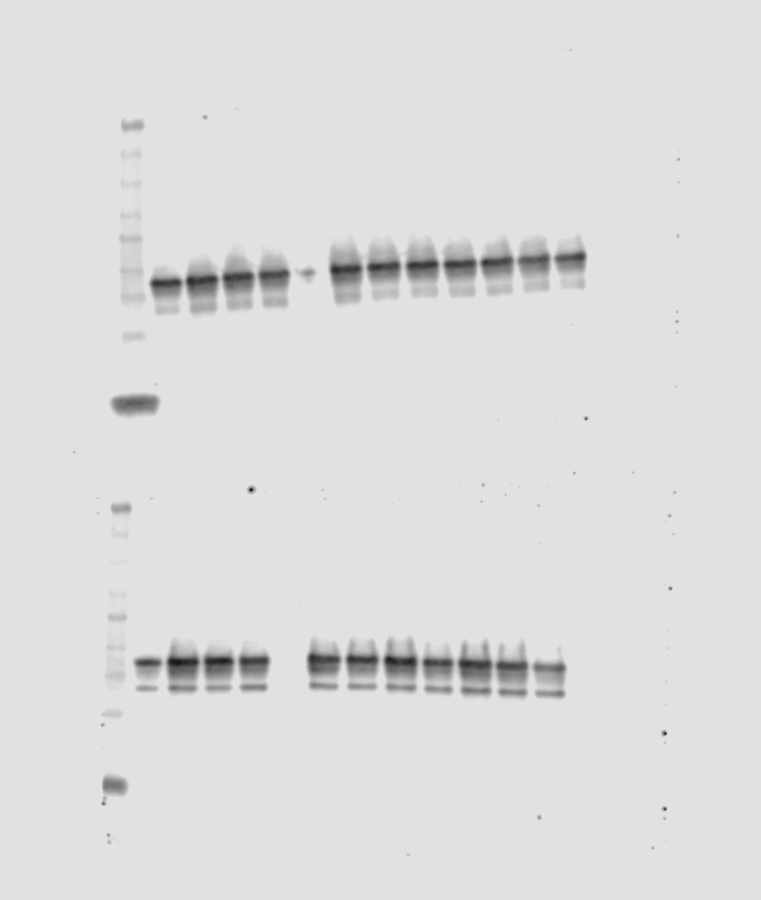


Additional raw blot of soluble β-actin and GAPDH (top) and insoluble β-actin and GAPDH (bottom) used for quantification in Fig 5G.


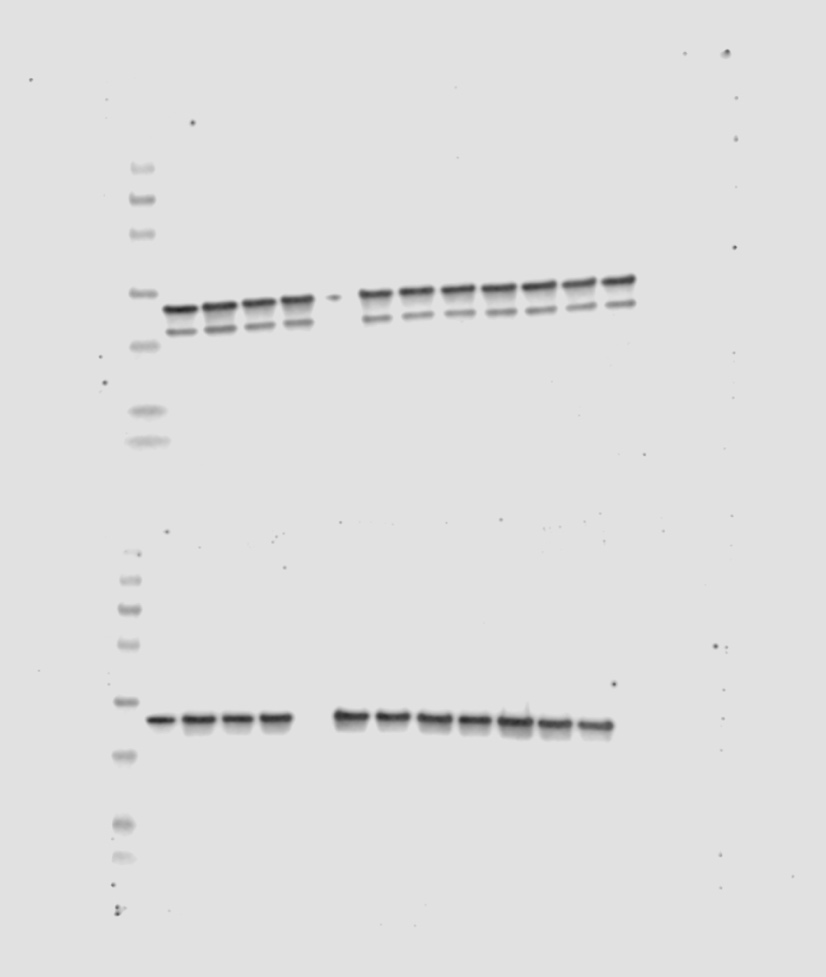


Raw blot of soluble pTDP-43 (S409/410) (sample lanes 4-9, top) and insoluble pTDP-43 (S409/410) (samples lanes 4-9, bottom) shown in Fig 5E and used for quantification in Fig 5H.


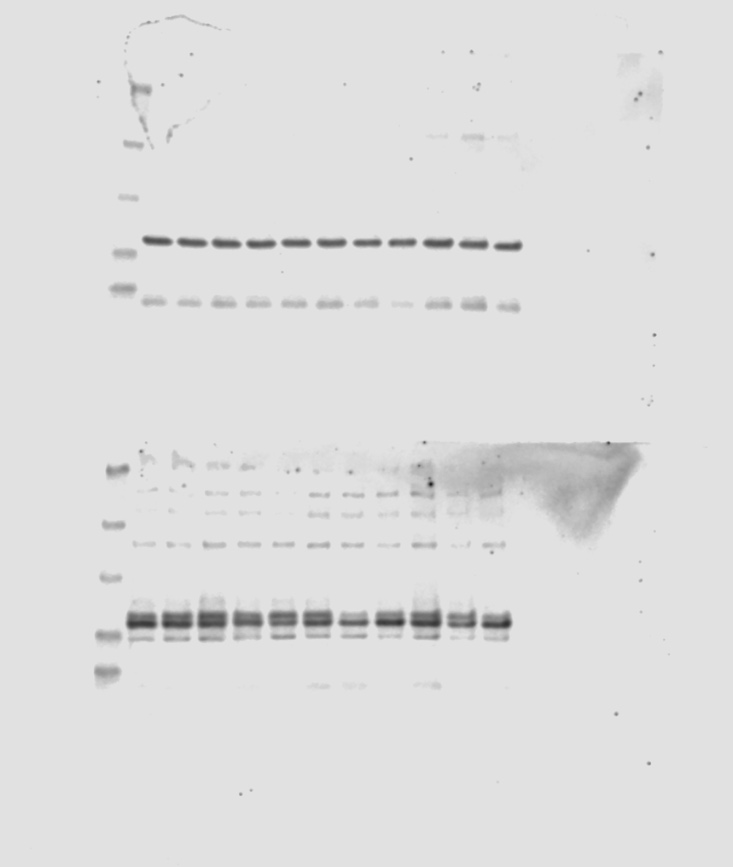


Raw blot of soluble TDP-43 (sample lanes 4-9, top) and insoluble TDP-43 (samples lanes 4-9, bottom) shown in Fig 5E and used for quantification in Fig 5H.


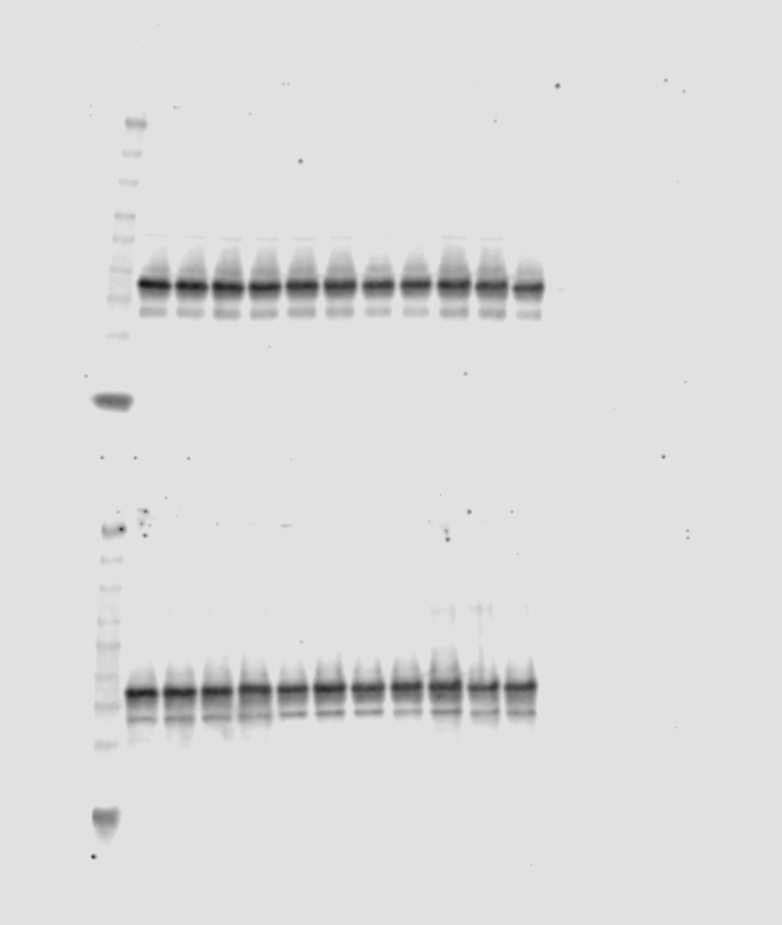


Raw blot of soluble β-actin and GAPDH (sample lanes 4-9, top) and insoluble β-actin and GAPDH (samples lanes 4-9, bottom) shown in Fig 5E and used for quantification in Fig 5H.


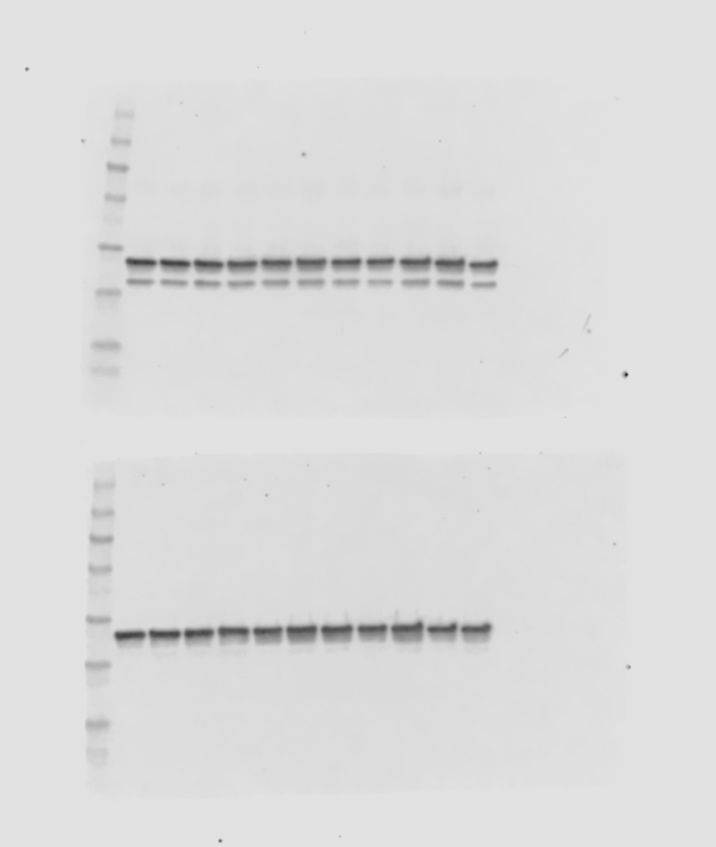


Additional raw blot of soluble pTDP-43 (S409/410) (sample lanes 2-7) and insoluble pTDP-43 (S409/410) (sample lanes 9-14) used for quantification in Fig 5H.


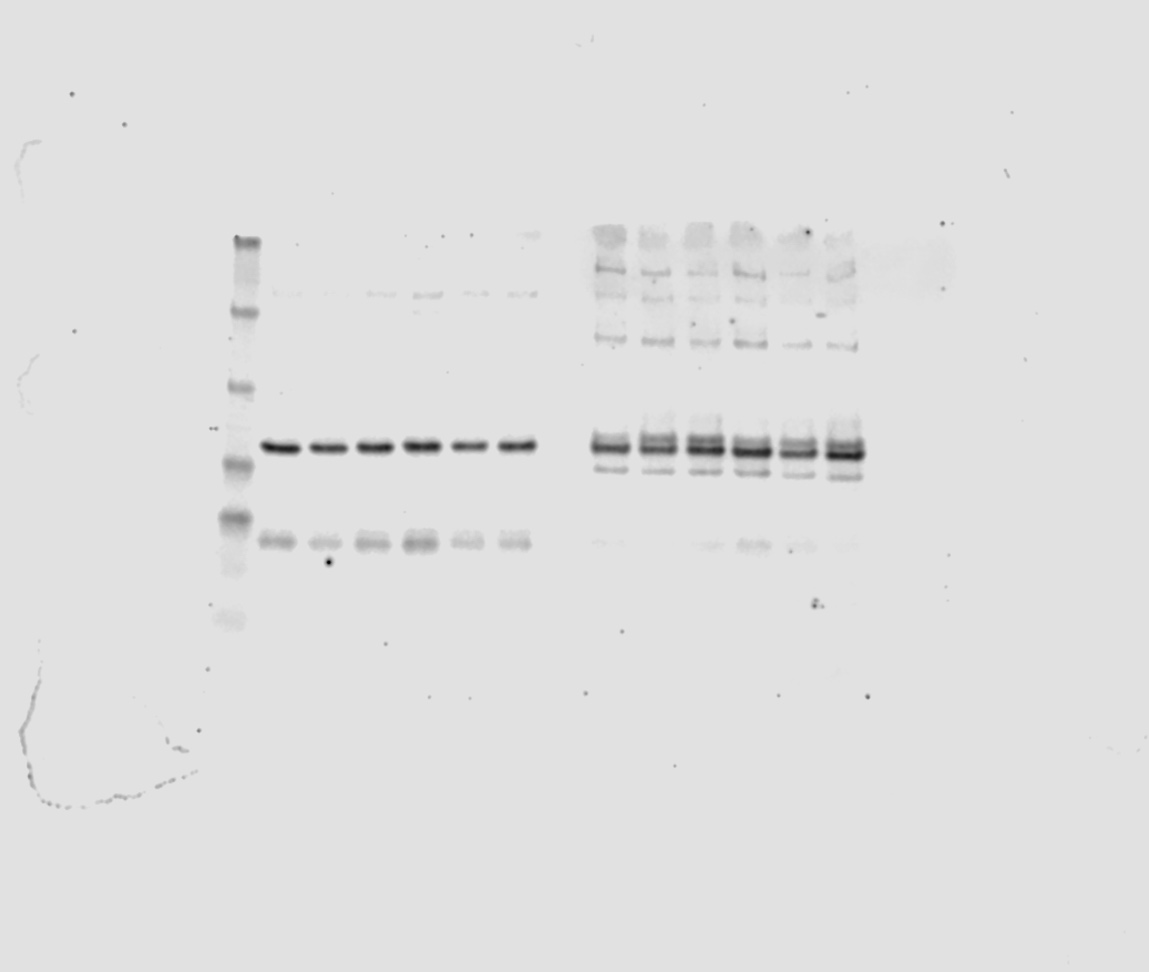


Additional raw blot of soluble TDP-43 (sample lanes 2-7) and insoluble TDP-43 (sample lanes 9-14) used for quantification in Fig 5H.


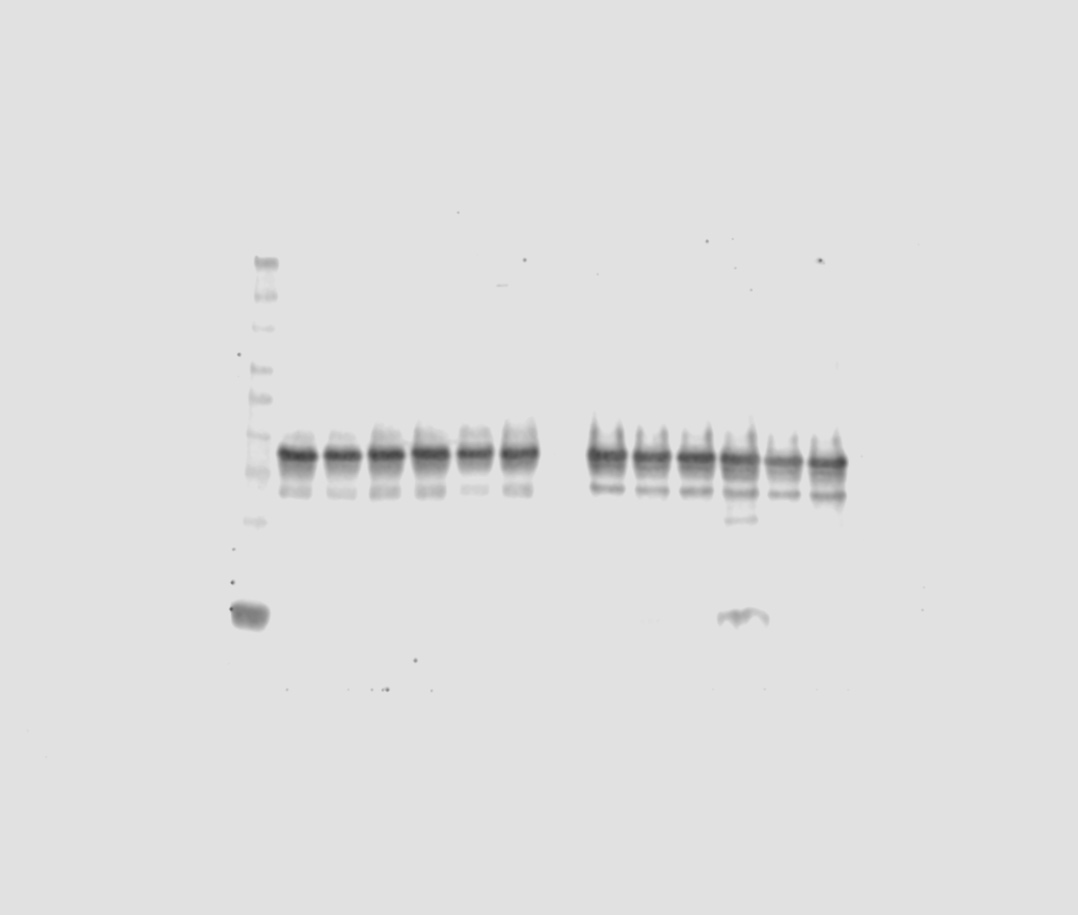


Additional raw blot of soluble β-actin and GAPDH (sample lanes 2-7) and insoluble β-actin and GAPDH (sample lanes 9-14) used for quantification in Fig 5H.


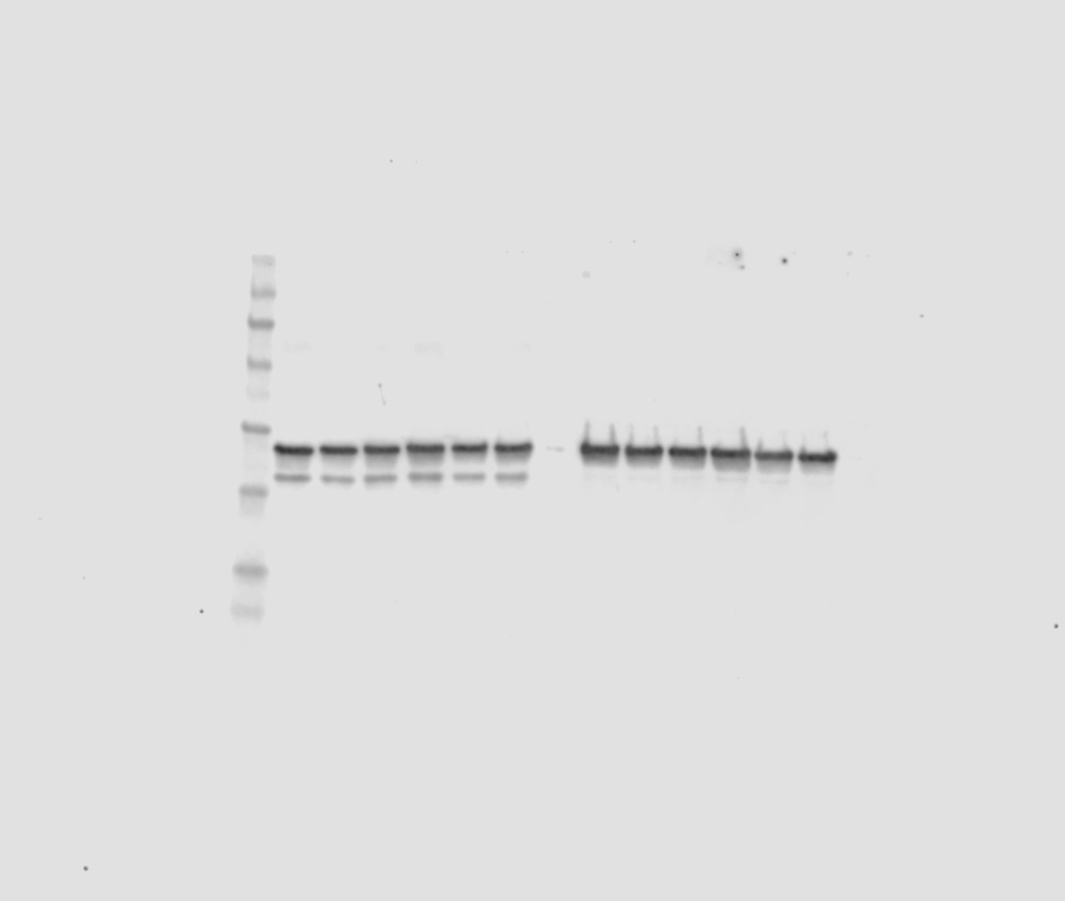


Raw blot of soluble pTDP-43 (S409/410) (sample lanes 6-11, top) and insoluble pTDP-43 (S409/410) (samples lanes 6-11, bottom) shown in Fig 5F and used for quantification in Fig 5I.


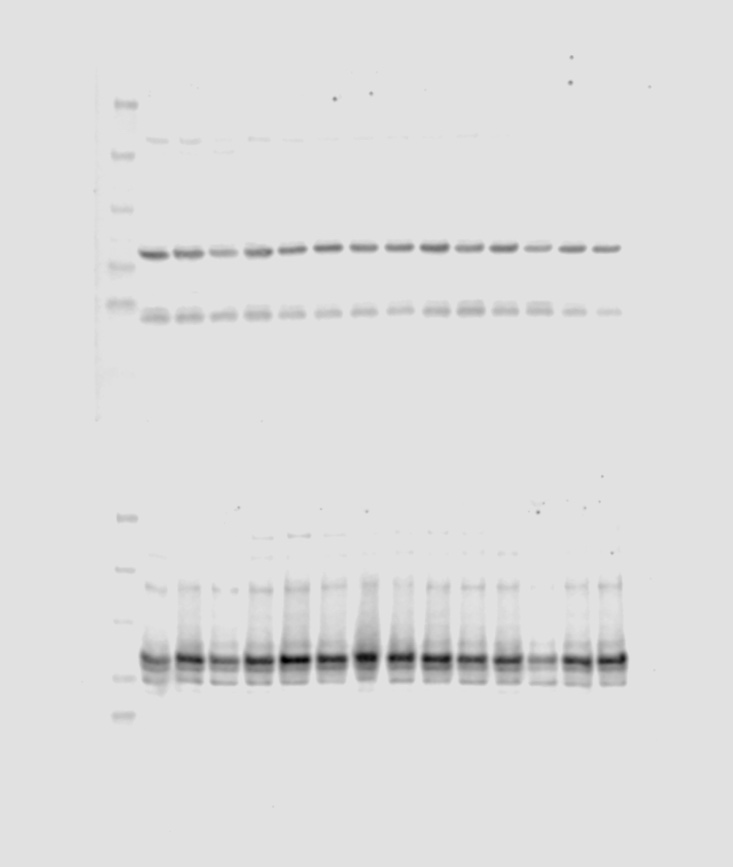


Raw blot of soluble TDP-43 (sample lanes 6-11, top) and insoluble TDP-43 (samples lanes 6-11, bottom) shown in Fig 5F and used for quantification in Fig 5I.


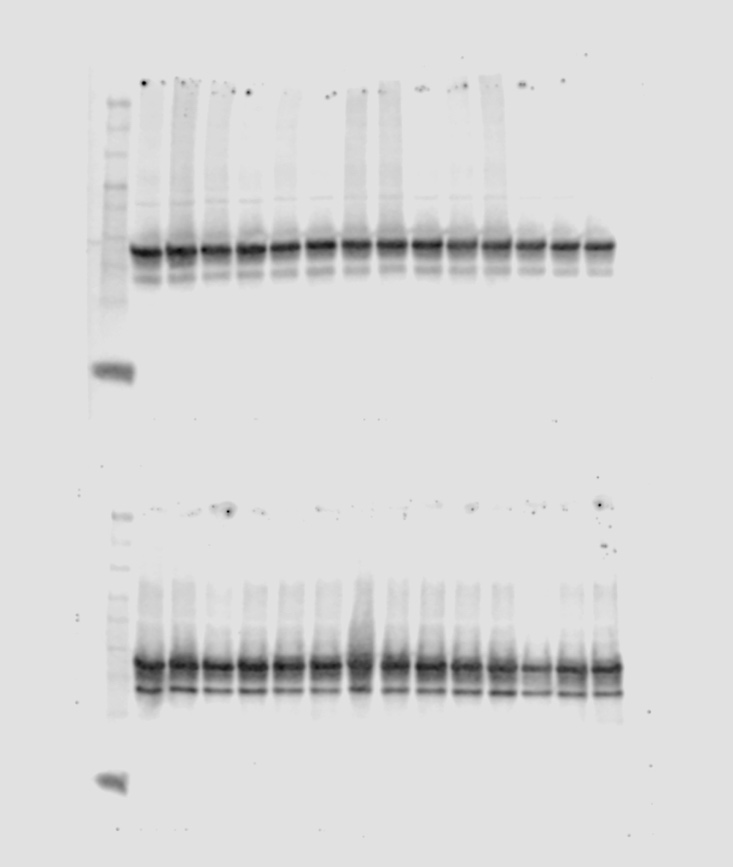


Raw blot of soluble β-actin and GAPDH (sample lanes 6-11, top) and insoluble β-actin and GAPDH (samples lanes 6-11, bottom) shown in Fig 5F and used for quantification in Fig 5I.


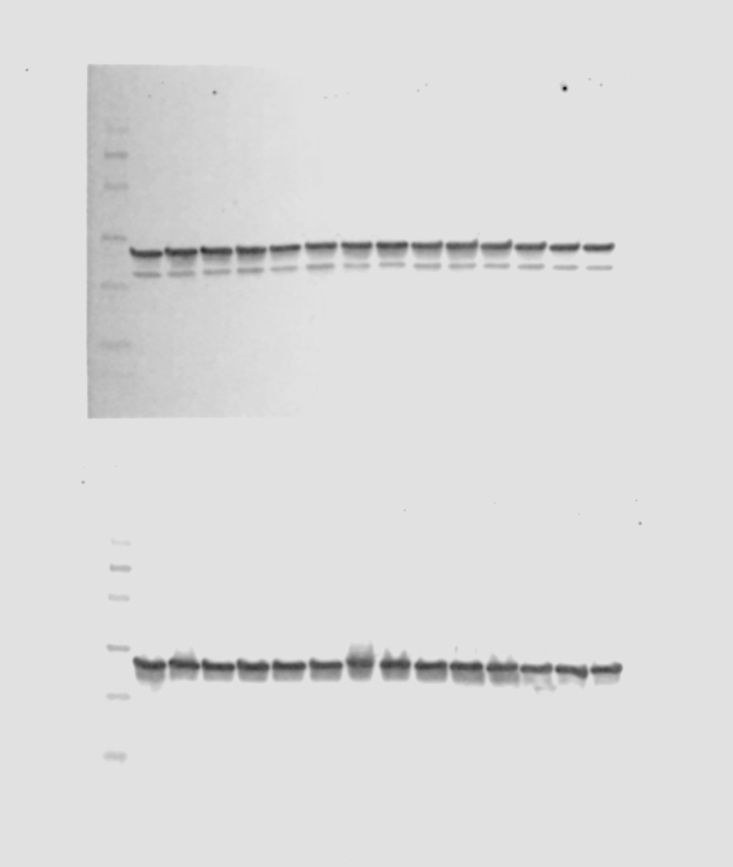


Raw blot of CK1ε (sample lanes 2-4, 6-7, 9-10, top) and CK1δ (sample lanes 2-4, 6-7, 9-10, bottom) shown in Supplementary Fig S1D and S1E.


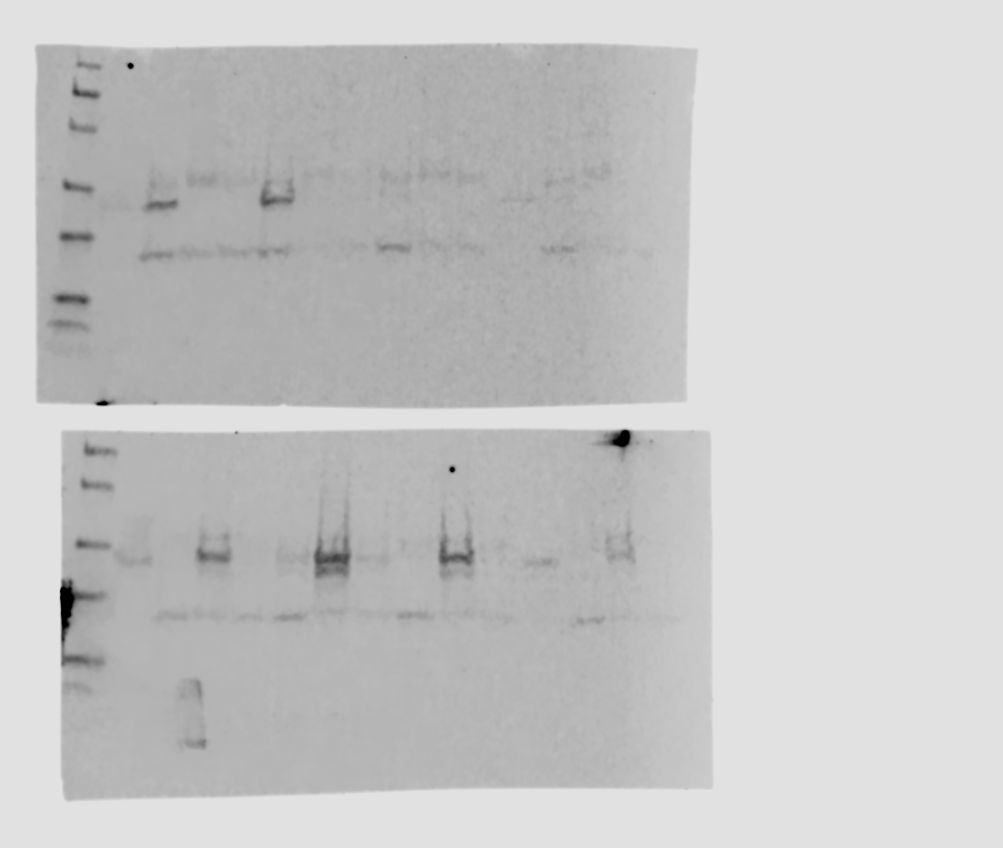


Raw blot of soluble pTDP-43 (S409/410) (sample lanes 2-7, top) and insoluble pTDP-43 (S409/410) (samples lanes 2-7, bottom) shown in Supplementary Fig S4A.


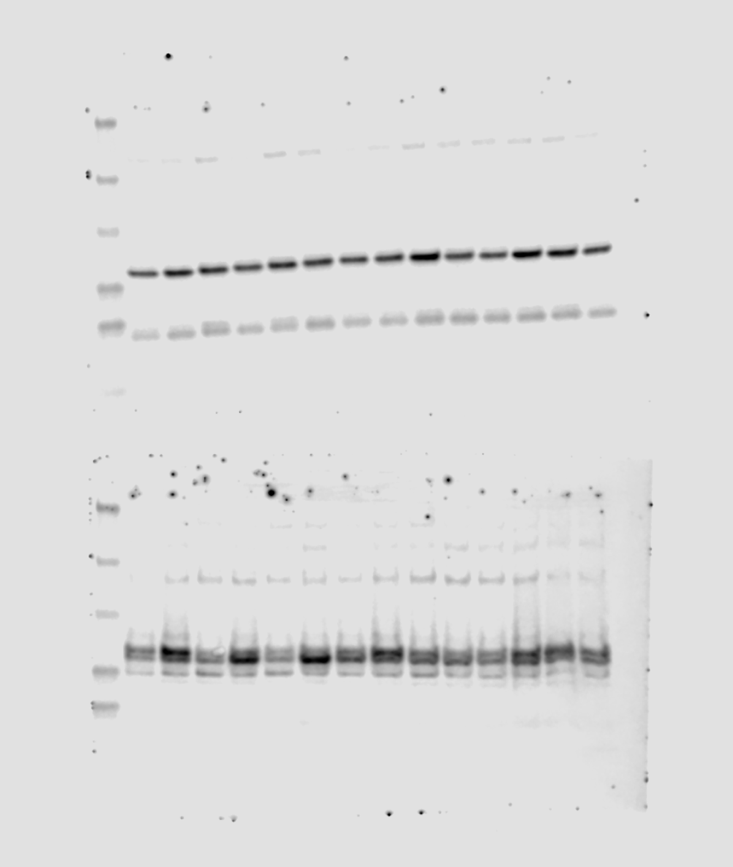


Raw blot of soluble TDP-43 (sample lanes 2-7, top) and insoluble TDP-43 (samples lanes 2-7, bottom) shown in Supplementary Fig S4A.


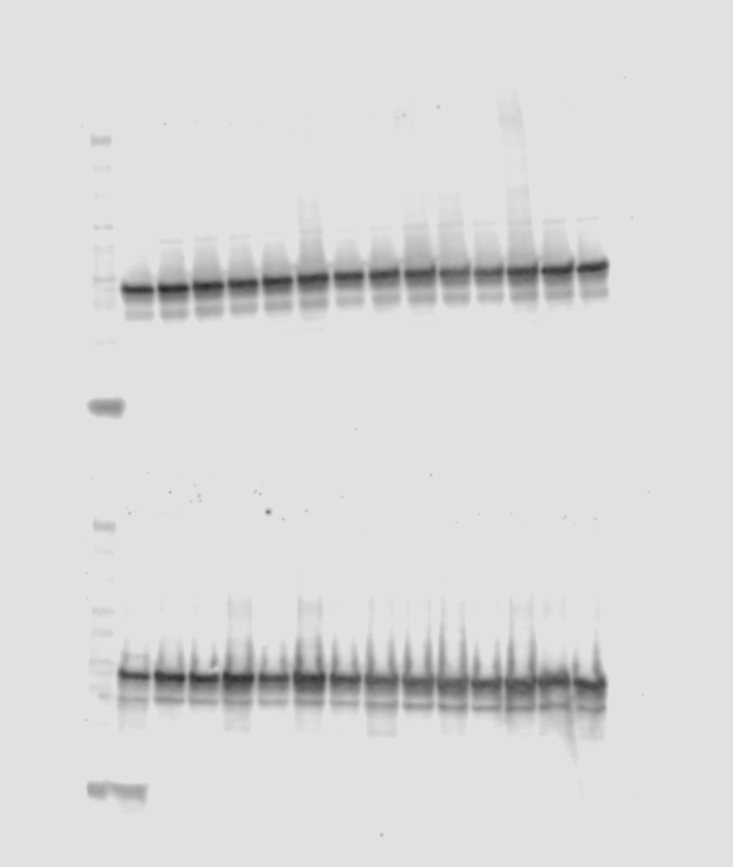


Raw blot of soluble β-actin and GAPDH (sample lanes 2-7, top) and insoluble β-actin and GAPDH (samples lanes 2-7, bottom) shown in Supplementary Fig S4A.


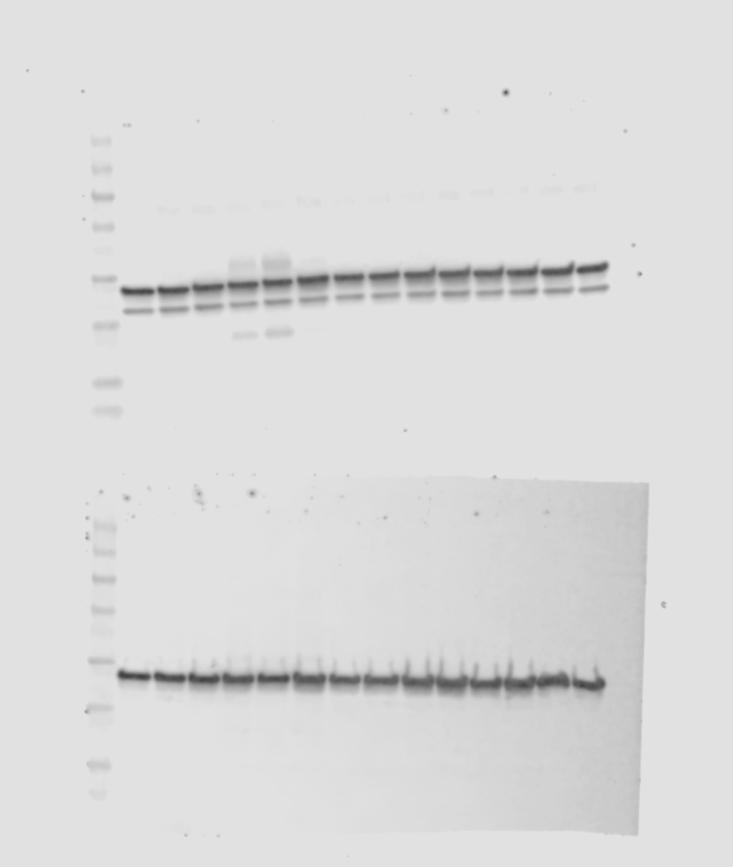

Supplement: Supplementary file 5 — Supplementary Material 5 [file 40478_2024_1902_MOESM5_ESM.docx]
