# Supplementary material for: CK1δ/ε-mediated TDP-43 phosphorylation contributes to early motor neuron disease toxicity in amyotrophic lateral sclerosis
Source: Acta Neuropathol Commun. 2024 Dec 4;12:187. doi: 10.1186/s40478-024-01902-z (PMC11619411; doi:10.1186/s40478-024-01902-z)
Supplement: Supplementary file 6 — Supplementary Material 6 [file 40478_2024_1902_MOESM6_ESM.docx]

**Supplemental Information File 1.**

**
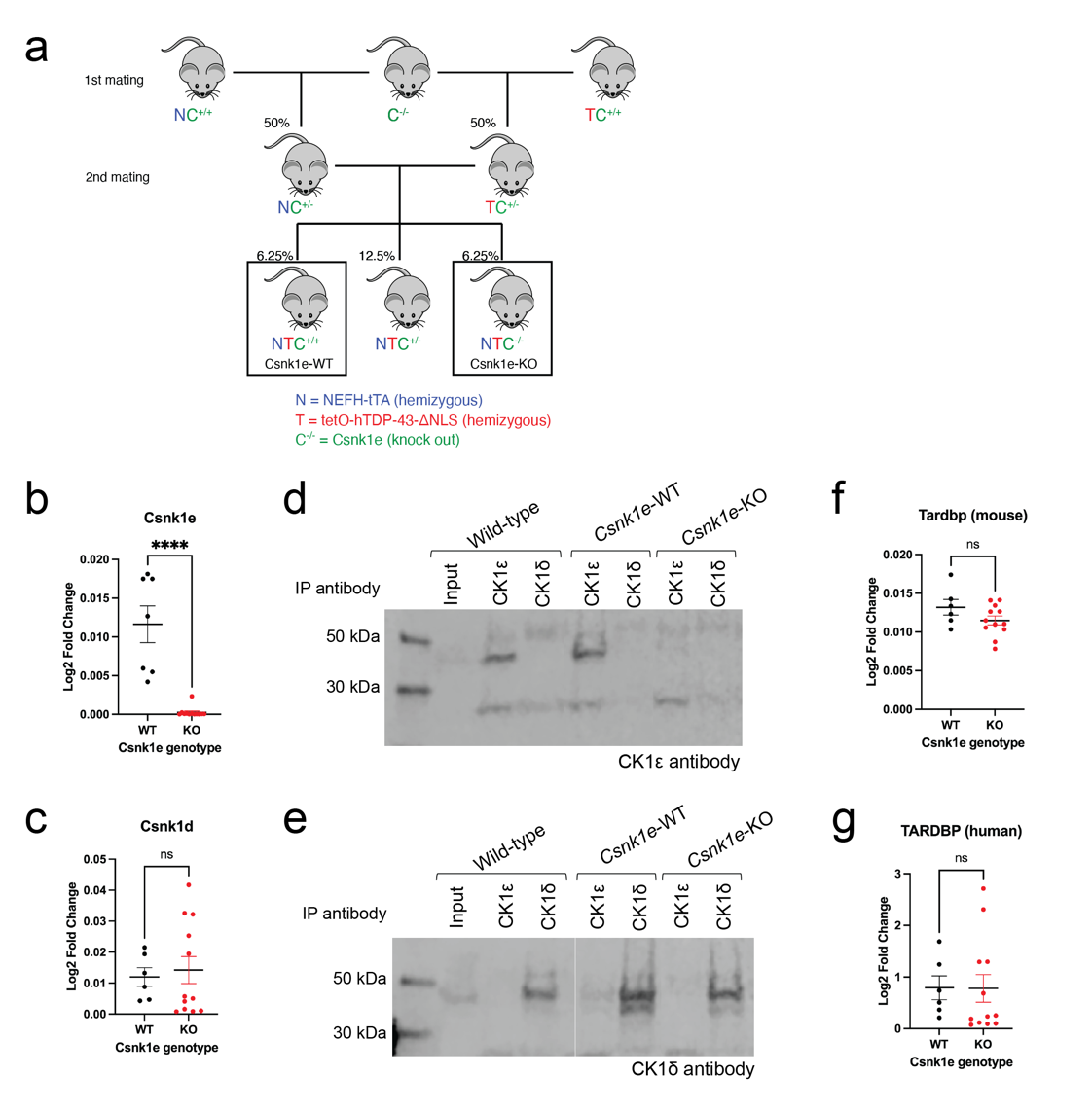
**

**Supplementary Figure S1. Breeding scheme and characterization of *Csnk1e*-KO x hTDP-43-ΔNLS.** (a) Breeding scheme used to generate *Csnk1e*-KO mice and *Csnk1e*-WT control litter mates. (b-c) qRT-PCR data of gene expression for *Csnk1e* and *Csnk1d* mRNA levels in mice. *GAPDH* mRNA was used as endogenous control. Deletion of *Csnk1e* in mice was successful, with no significant effect on *Csnk1d* gene expression. Mann-Whitney U test, *p < 0.03, **p < 0.002, ***p < 0.0002, ****p < 0.0001. Error bars represent SEM. (d-e) Immunoblots of brain cortex lysate after immunoprecipitation (due to low expression levels of CK1ε and CK1δ) showed *Csnk1e* specific knockout in mice where CK1ε was not detected in *Csnk1e*-KO samples (D) but CK1δ could still be detected (E). (f-g) qRT-PCR data of gene expression for mouse-specific *Tardbp* and human-specific *TARDBP* mRNA levels in mice. *GAPDH* mRNA was used as endogenous control. Deletion of *Csnk1e* in mice resulted in no significant effect on TDP-43 gene expression. Mann-Whitney U-test, *p < 0.03, **p < 0.002, ***p < 0.0002, ****p < 0.0001. Error bars represent SEM.


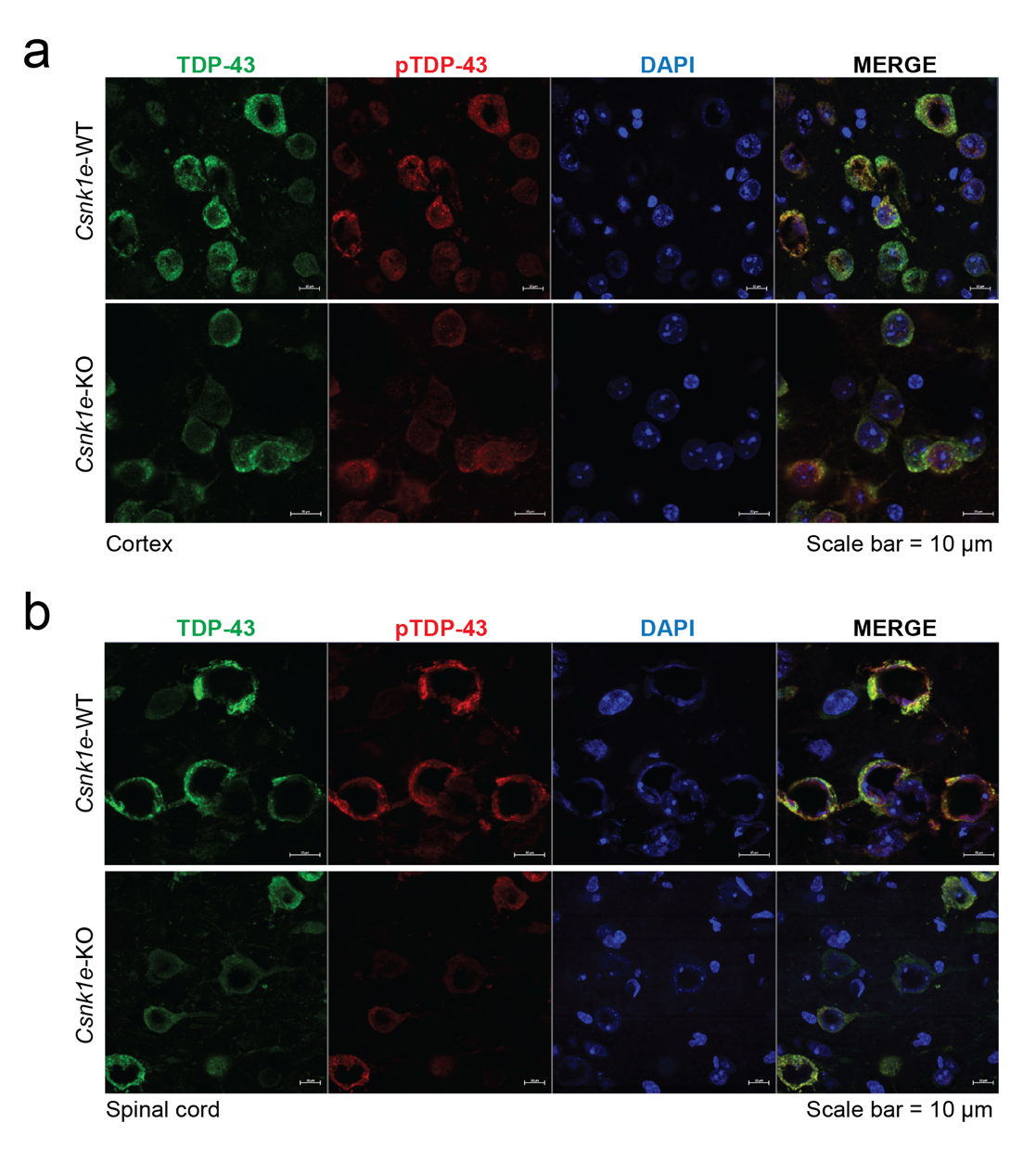


**Supplementary Figure S2.** ***Csnk1e*-KO mice developed cellular hallmarks of TDP-43 nuclear clearance and cytoplasmic phosphorylated TDP-43 expression in the brain and spinal cord.** (a-b) Representative immunofluorescence images of cortex and spinal cord tissue with TDP-43 (green), pTDP-43 (red), and DAPI (blue). No difference was observed in neurons in brain or spinal cord tissue from *Csnk1e*-KO mice as compared to *Csnk1e*-WT control mice. Scale bars represent 10 μm.

|  | **PF-05236216**  CK1δ/ε-selective | | | **PF-4800567**  CK1ε-selective | |
| --- | --- | --- | --- | --- | --- |
| **Chemical structure** | 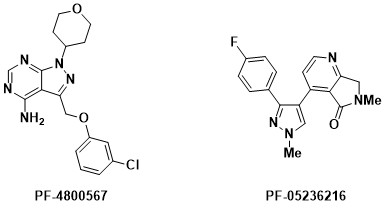 | | | 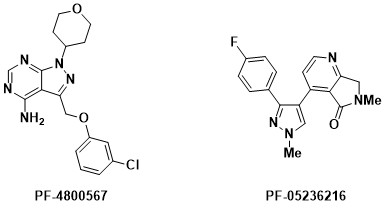 | |
| **CK1δ IC_50_ (nM)** | 8 [3] | | | 711 [1, 2] | |
| **CK1ε IC_50_ (nM)** | 36 [3] | | | 32 [1, 2] | |
|  | **Plasma** | **Brain*** | **Plasma** | | **Brain*** |
| **AUC (hr*ng/mL)** | 49909 | 22344 | 7996 | | 7490 |
| **Mean C_max_ (ng/mL)** | 17483 | 8447 | 5523 | | 5050 |
| **Mean terminal t_1/2_ (hr)** | 2 | 2.5 | 3.2 | | 6.7 |

* Concentration in brain is in ng/g

**Supplementary Table S1. CK1 inhibitor compound profiles.**

Additional characteristics of the CK1 inhibitor compounds are outlined. The chemical structure was made using ChemDraw.


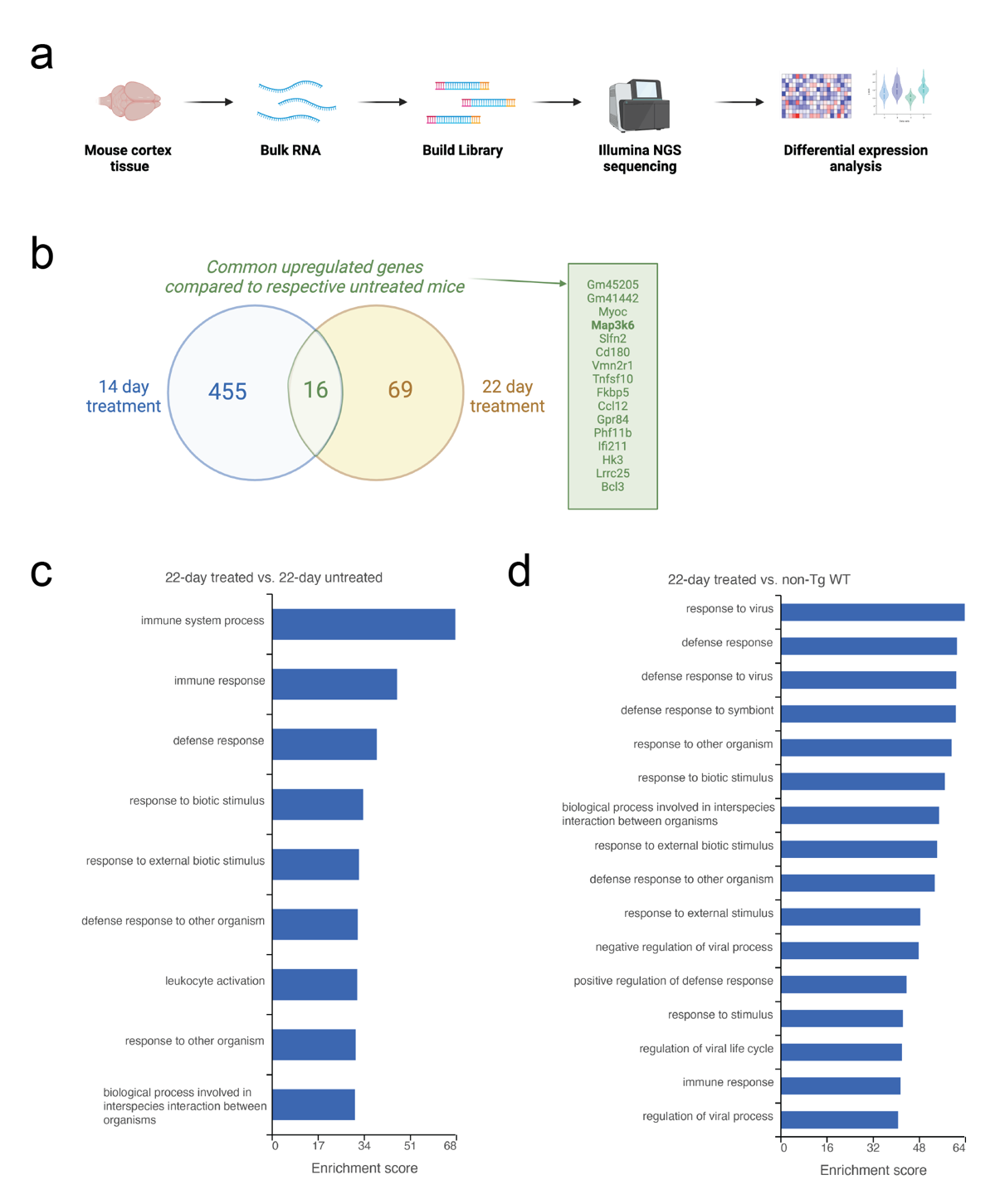


**Supplementary Figure S3.** **Transcriptomic study revealed potential side effects of long-term CK1δ/ε-selective inhibitor treatment.** (a) Schematic of experimental design to perform bulk-RNA sequencing of samples from the 14-day and 22-day treated and untreated mice groups. (b) Venn diagram comparing highly differentiated genes between treated and untreated mice for 14 and 22 days. *MAP3K6* was identified to be significantly upregulated in the treated groups compared to untreated groups at both timepoints. (c-d) Gene set enrichment analysis (GSEA) of 22-day treated, 22-day untreated, and non-Tg WT mice did not identify any significant differences between the gene ontology groups and enrichment scores.


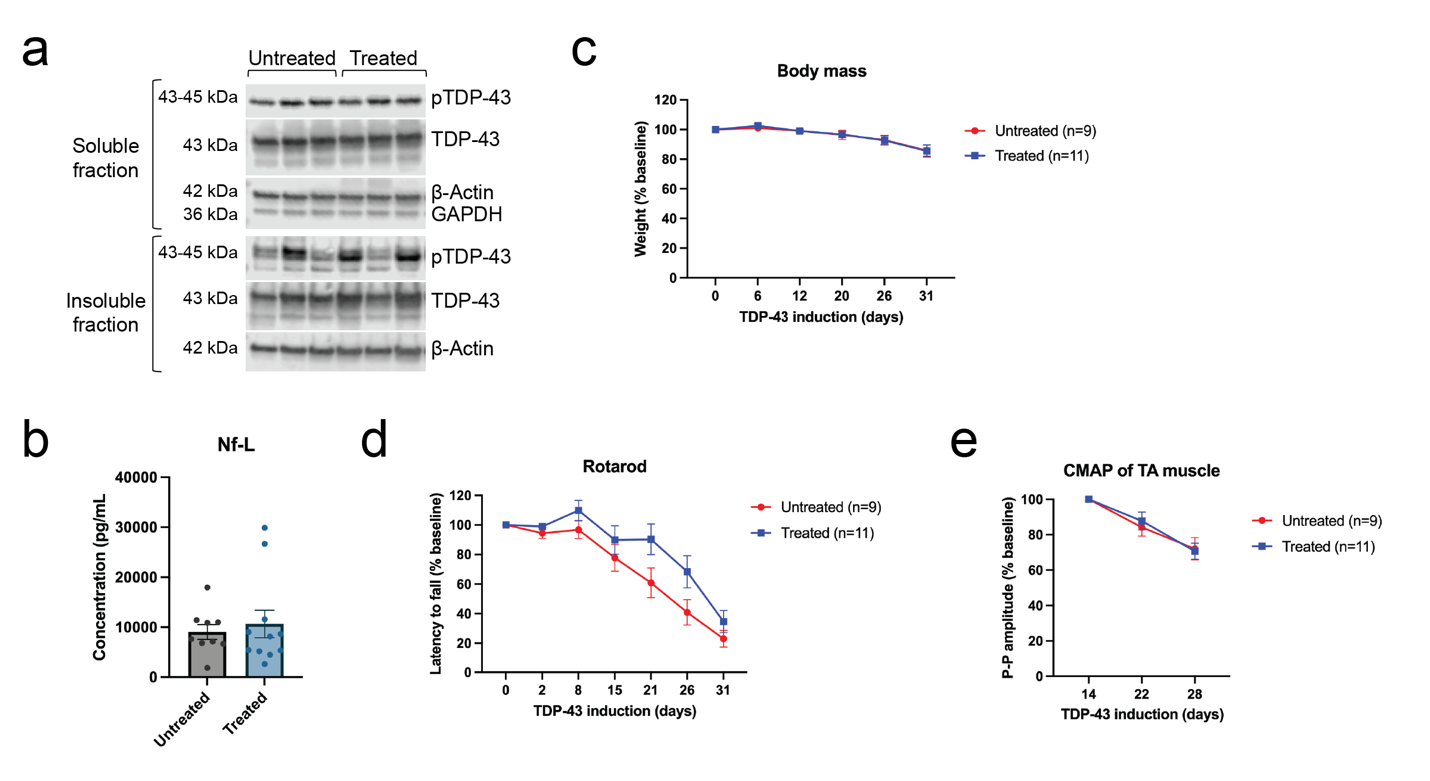


**Supplementary Figure S4. Analysis of alternative dosing route via continuous chow infusion showed comparable results as that of daily single IP dosing.** (a) Immunoblots of untreated mice and mice treated with 20 mg/kg CK1δ/ε-specific inhibitor PF-05236216 in chow format. Samples are of brain cortex collected on the last day, end of the 31-day study. There was no difference in pTDP-43 levels detected in both soluble and insoluble protein fractions between untreated and treated groups. (b-e) The cohort for Nf-L, body mass, rotarod, and CMAP included 9 untreated hTDP-43-ΔNLS mice (3 male, 6 female) and 11 treated hTDP-43-ΔNLS mice (5 male, 6 female). No significant difference was detected between treatment groups throughout the study for Nf-L (b), body mass (c), rotarod (d), or CMAP of TA muscle (e).

**References**

1. Cozza G, Pinna LA (2016) Casein kinases as potential therapeutic targets. Expert Opin Ther Targets 20:319–340. doi: 10.1517/14728222.2016.1091883

2. Li S-S, Dong Y-H, Liu Z-P (2021) Recent Advances in the Development of Casein Kinase 1 Inhibitors. Curr Med Chem 28:1585–1604. doi: 10.2174/0929867327666200713185413

3. Wager TT, Galatsis P, Chandrasekaran RY, Butler TW, Li J, Zhang L, Mente S, Subramanyam C, Liu S, Doran AC, Chang C, Fisher K, Grimwood S, Hedde JR, Marconi M, Schildknegt K (2017) Identification and Profiling of a Selective and Brain Penetrant Radioligand for in Vivo Target Occupancy Measurement of Casein Kinase 1 (CK1) Inhibitors. ACS Chem Neurosci 8:1995–2004. doi: 10.1021/acschemneuro.7b00155
